# Nucleolar detention of NONO shields DNA double-strand breaks from aberrant transcripts

**DOI:** 10.1093/nar/gkae022

**Published:** 2024-01-15

**Authors:** Barbara Trifault, Victoria Mamontova, Giacomo Cossa, Sabina Ganskih, Yuanjie Wei, Julia Hofstetter, Pranjali Bhandare, Apoorva Baluapuri, Blanca Nieto, Daniel Solvie, Carsten P Ade, Peter Gallant, Elmar Wolf, Dorthe H Larsen, Mathias Munschauer, Kaspar Burger

**Affiliations:** Mildred Scheel Early Career Center for Cancer Research (Mildred-Scheel-Nachwuchszentrum, MSNZ) Würzburg, University Hospital Würzburg, Josef-Schneider-Strasse 2, D-97080 Würzburg, Germany; Department of Biochemistry and Molecular Biology, Biocenter of the University of Würzburg, Am Hubland, D-97074 Würzburg, Germany; Mildred Scheel Early Career Center for Cancer Research (Mildred-Scheel-Nachwuchszentrum, MSNZ) Würzburg, University Hospital Würzburg, Josef-Schneider-Strasse 2, D-97080 Würzburg, Germany; Department of Biochemistry and Molecular Biology, Biocenter of the University of Würzburg, Am Hubland, D-97074 Würzburg, Germany; Department of Biochemistry and Molecular Biology, Biocenter of the University of Würzburg, Am Hubland, D-97074 Würzburg, Germany; Helmholtz Institute for RNA-based Infection Research, Helmholtz-Center for Infection Research, Josef-Schneider-Strasse 2, D-97080 Würzburg, Germany; Helmholtz Institute for RNA-based Infection Research, Helmholtz-Center for Infection Research, Josef-Schneider-Strasse 2, D-97080 Würzburg, Germany; Cancer Systems Biology Group, Theodor Boveri Institute, Biocenter, University of Würzburg, Am Hubland, D-97074 Würzburg, Germany; Cancer Systems Biology Group, Theodor Boveri Institute, Biocenter, University of Würzburg, Am Hubland, D-97074 Würzburg, Germany; Cancer Systems Biology Group, Theodor Boveri Institute, Biocenter, University of Würzburg, Am Hubland, D-97074 Würzburg, Germany; Nucleolar Stress and Disease Group, Danish Cancer Institute, Strandboulevarden 49, Copenhagen, Denmark; Department of Biochemistry and Molecular Biology, Biocenter of the University of Würzburg, Am Hubland, D-97074 Würzburg, Germany; Department of Biochemistry and Molecular Biology, Biocenter of the University of Würzburg, Am Hubland, D-97074 Würzburg, Germany; Department of Biochemistry and Molecular Biology, Biocenter of the University of Würzburg, Am Hubland, D-97074 Würzburg, Germany; Cancer Systems Biology Group, Theodor Boveri Institute, Biocenter, University of Würzburg, Am Hubland, D-97074 Würzburg, Germany; Nucleolar Stress and Disease Group, Danish Cancer Institute, Strandboulevarden 49, Copenhagen, Denmark; Helmholtz Institute for RNA-based Infection Research, Helmholtz-Center for Infection Research, Josef-Schneider-Strasse 2, D-97080 Würzburg, Germany; Mildred Scheel Early Career Center for Cancer Research (Mildred-Scheel-Nachwuchszentrum, MSNZ) Würzburg, University Hospital Würzburg, Josef-Schneider-Strasse 2, D-97080 Würzburg, Germany; Department of Biochemistry and Molecular Biology, Biocenter of the University of Würzburg, Am Hubland, D-97074 Würzburg, Germany

## Abstract

RNA-binding proteins emerge as effectors of the DNA damage response (DDR). The multifunctional non-POU domain-containing octamer-binding protein NONO/p54^nrb^ marks nuclear paraspeckles in unperturbed cells, but also undergoes re-localization to the nucleolus upon induction of DNA double-strand breaks (DSBs). However, NONO nucleolar re-localization is poorly understood. Here we show that the topoisomerase II inhibitor etoposide stimulates the production of RNA polymerase II-dependent, DNA damage-inducible antisense intergenic non-coding RNA (asincRNA) in human cancer cells. Such transcripts originate from distinct nucleolar intergenic spacer regions and form DNA–RNA hybrids to tether NONO to the nucleolus in an RNA recognition motif 1 domain-dependent manner. NONO occupancy at protein-coding gene promoters is reduced by etoposide, which attenuates pre-mRNA synthesis, enhances NONO binding to pre-mRNA transcripts and is accompanied by nucleolar detention of a subset of such transcripts. The depletion or mutation of NONO interferes with detention and prolongs DSB signalling. Together, we describe a nucleolar DDR pathway that shields NONO and aberrant transcripts from DSBs to promote DNA repair.

## Graphical Abstract

**Graphical Abstract:**
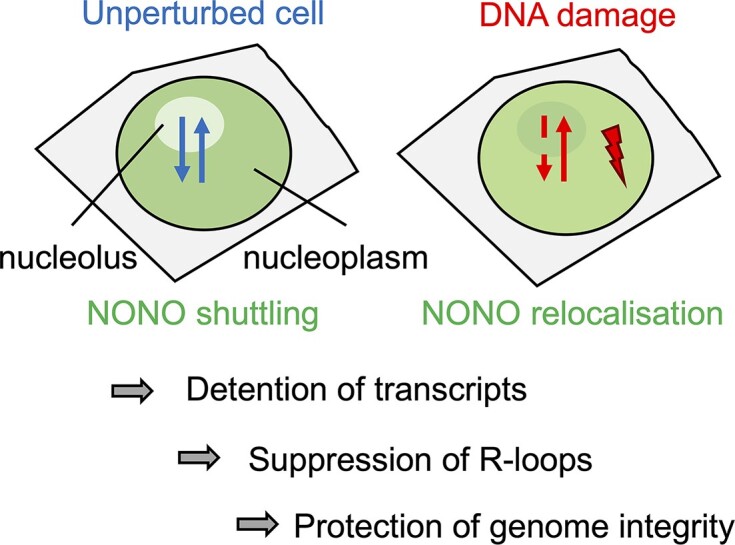

## Introduction

Genome stability requires faithful inheritance of genetic information. The DNA damage response (DDR) recognizes and repairs DNA lesions to maintain genome stability (1–3). Unscheduled RNA synthesis exposes DNA, which can lead to deleterious DNA double-strand breaks (DSBs) (4,5). Thus, the bulk of transcription is impaired during DSB repair (6). DSB-sensing kinases such as *Ataxia-telangiectasia mutated* (ATM) activate > 100 factors to catalyse DSB repair via homologous recombination (HR) or non-homologous end-joining (NHEJ) (7). However, 40% of DNA damage-induced phosphorylation events address factors related to nucleic acid metabolism, in particular RNA-binding proteins (RBPs) (8–10). Thus, RNA metabolism emerges as a stimulator of genome maintenance (11–13). The members of the *Drosophila* behaviour/human splicing (DBHS) protein family (non-POU domain-containing octamer-binding protein, NONO; splicing factor proline and glutamine rich, SFPQ; and paraspeckle component 1, PSPC1) are multifunctional RBPs that dynamically associate with sites of active transcription and nuclear bodies called paraspeckles, but also participate in genome maintenance (14–18). NONO binds DNA ends and promotes the activation of DNA-dependent protein kinase (DNA-PK) to stimulate DSB repair (19–23). Interestingly, NONO accumulates in condensates induced by transcription inhibition, which are associated with disintegrated nucleoli and mitigate the formation of aberrant gene fusions (24), and is also enriched in non-disintegrated nucleoli upon DNA damage (25). However, the relevance of NONO nucleolar re-localization for DDR remains elusive.

Here we show that the DNA topoisomerase II inhibitor etoposide induces RNA polymerase II (RNAPII)-dependent nucleolar antisense transcripts, which form DNA–RNA hybrids (R-loops) at nucleolar intergenic spacer (IGS) loci to deplete NONO from protein-coding gene promoters. NONO re-localization reduces pre-mRNA synthesis and detains pre-mRNA transcripts, which fosters efficient DSB signalling. Our data suggest a nucleolar DDR pathway that engages DNA damage-induced nucleolar RNAPII activity in genome maintenance.

## Materials and methods

### Tissue culture

Human U2OS, AsiSI-ER-expressing U2OS (DIvA, kind gift from Gaelle Legube), green fluorescent protein (GFP)–APEX2-NIK3-expressing U2OS (U2OS:GFP-APEX2-NIK3) and HEK293 cells were cultured in Dulbecco's modified Eagle's medium (DMEM, Gibco) with 10% fetal bovine serum (FBS, Capricorn), 100 U/ml penicillin–streptomycin (Gibco), 2 mM l-glutamine (Gibco) at 37°C and 5% CO_2_. Cells were incubated with etoposide (Sigma, 20 μM), neocarzinostatin (NCS; Sigma, 500 ng/ml), KU-55933 (Hycultec, 1 μM), THZ1 (Biozol, 1 μM) for 2 h and 4-hydroxytamoxifen (4-OHT; Sigma, 10 μM) for 4 h, unless stated otherwise.

### Transfection and viral work

Transfection of small-interfering RNA (siRNA; 100 nM) or plasmids pBABe:I-PpoI-ER (kind gift from Michael Kastan), pBABe:AsiSI-ER (kind gift from Gaelle Legube), pEGFP-RNaseH1 (kind gift from Martin Reijns), ppyCAG-V5-RNaseH1 and ppyCAG-V5-RNaseH1 D210N (kind gifts from Xiang-Dong Fu), pmCherry-NONO (kind gift from Ling-Ling Chen) and pcDNA3.1-HA-NONO (kind gift from Nicolas Manel) was performed using Lipofectamine 2000 (Invitrogen) and Opti-MEM (Gibco) following the manufacturer's protocol. HA-NONO mutants were cloned with selective primers (Supplementary Table S1) and a Q5 site-directed mutagenesis kit (NEB) following the manufacturer's protocol and verified by Sanger sequencing. For transfection with oligonucleotides, cells were transfected (6 h) on two consecutive days with siRNA (100 nM) (Supplementary Table S2) or 1 day with gapmers (100 nM). For short hairpin RNA (shRNA) depletion, cells were transduced with shRNA targeting NONO (pool of pLKO.1-puro-NONO-sh1, pLKO.1-puro-NONO-sh5, kind gift from Nicolas Manel) or non-targeting control (pLKO.1-puro-non-target shRNA, shCtrl., Sigma) by lentiviral infection. To generate U2OS:GFP-APEX2-NIK3 cells, 10 μg of pLX304-GFP-APEX2-NIK3 plasmid (kind gift from Alice Ting) was pooled with psPAX2 and pMD2.G (kind gift from Elmar Wolf), mixed with 30 μl of polyethylenimine (Calbiochem), diluted in 500 μl of OptiMEM, vortexed, incubated (25 min at room temperature), added to HEK293 cells that were pre-incubated in 5 ml of DMEM/2% FBS, and transfected (8 h). Virus was harvested three times every 12 h, sterile filtered and frozen. For infection, U2OS cells were cultured (24 h) in viral mixture (1.5 ml of DMEM, 1.5 ml of viral harvest, 6 μl of polybrene, Invitrogen). The mixture was replaced by DMEM with 7.5 μg/ml blasticidin (Sigma) for polyclonal selection (10 days). AlamarBlue viability assay (Thermo) was performed following the manufacturer's protocol. Resorufin was quantified with a plate reader (TECAN).

### Ribonucleoprotein transfection

DSBs were induced by transfection of ribonucleoprotein (RNP) complexes, consisting of purified recombinant Cas9 protein (TrueCut Cas9 protein v2, #36499, Thermo) and synthetic guide RNA (Trueguide sgRNA #35514 or Trueguide sgRNA negative control, non-targeting 1, #A35526, Thermo) using Lipofectamine CRISPRMAX Cas9 transfection reagent (#CMAX00015, Thermo) according to the manufacturer’s protocol, but with 25% of the recommended amount of Cas9, sgRNA and Cas9 Plus reagent, keeping the stoichiometric ratio as recommended. For locus-specific induction of DSBs in 5S DNA, the sgRNA sequence GUCCGAGAUCAGACGAGAUC was used. For induction of DSBs in rDNA, a combination of sgRNA 1 [CGAGAGAACAGCAGGCCCGC, targets the 5′ external transcribed spacer (5′ETS)] and sgRNA 3 (GAUUUCCAGGGACGGCGCCU, targets the IGS) was used in a 1:1 molar ratio.

### Fluorescence-activated cell sorting (FACS)

Cells were washed in phosphate-buffered saline (PBS), trypsinized, resuspended in DMEM and centrifuged (1500 rpm, 5 min, 4°C). Pellets were washed in PBS, centrifuged (1500 rpm, 5 min, 4°C), resuspended in 1 ml of PBS and fixed in 4 ml ice-cold 100% ethanol (−20°C, overnight). Cells were pelleted (1500 rpm, 10 min), washed in PBS, pelleted again and resuspended in 1 ml of PBS. A total of 1 × 10^6^ cells were stained with 54 μM propidium iodide (Sigma) in the presence of 24 μg/ml RNase A (Sigma) (30 min, 37°C, dark), sorted and analysed by a FACSDiva 9.0.1 flow cytometer (50 000 events per condition) and software (BD Biosciences).

### Immunoblotting and immunoprecipitation

Proteins were assessed as whole-cell extracts, directly lysed, boiled and sonicated in 4× sample buffer [250 mM Tris–HCl pH 6.8, 8% sodium dodecyl sulphate (SDS), 40% glycerol, 0.8% β-mercaptoethanol, 0.02% bromophenol blue]. Samples were separated by SDS–polyacrylamide gel electrophoresis (PAGE), transferred to nitrocellulose membranes (Cytiva), stained with 0.5% Ponceau S/1% acetic acid, blocked, washed in PBS/0.1% Triton X-100/5% milk (PBST), probed with selective antibodies (Supplementary Table S3) and visualized with an ECL kit (Cytiva) and an imaging station (LAS-4000, Fuji or Fusion FX, Vilber). Signals were quantified by ImageJ (NIH). For immunoprecipitation (IP), cells were trypsinized, washed in PBS and centrifuged (1200 rpm, 5 min). Pellets were lysed (10 min on ice) in 5 vols of IP buffer (200 mM NaCl, 0.5 mM EDTA, 20 mM HEPES, 0.2% NP-40, 10% glycerol, 400 U Ribolock inhibitor, 1× protease/phosphatase inhibitor, Roche). Lysates were centrifuged (12 000 rpm, 12 min) and supernatants were incubated (2 h, 4°C) with 2–5 μg of primary antibodies, pre-conjugated to 25 μl of Protein G Dynabeads (Invitrogen). Immunocomplexes were immobilized on a magnet (Invitrogen), washed three times with 800 μl of IP buffer (10 min, 4°C) and eluted with sample buffer (5 min, 95°C).

### Pull-down assays

Gapmers (100 pmol) (Supplementary Table S4) were stained with a SilverQuest kit (Invitrogen) or labelled with biotin-16-ddUTP (Jena Bioscience) and a second-generation DIG-oligonucleotide 3′end-labelling kit (Roche) following the manufacturer's protocol or with radioactive labelling mix [1 μl of 10× PNK buffer (NEB), 1 μl of 100 μM gapmer, 1 μl of T4 PNK (NEB), 1 μl of [γ-^32^P]ATP (Hartmann) and 6 μl of ddH_2_O] for 40 min at 37°C. End-labelled gapmers were centrifuged (3200 rpm, 5 min) with G-25 columns (Cytiva), diluted in 800 μl of IP buffer and incubated (2 h at room temperature with rotation) with either 0.4 μg of recombinant NONO (rec-NONO) (ActiveMotif) or HA-NONO variants that were immobilized on HA-conjugated Protein G Dynabeads (Invitrogen) upon expression in HEK293 cells and IP from whole-cell lysates. Rec-NONO complexes were captured on 25 μl of streptavidin C1 Dynabeads (Invitrogen), washed twice with 800 μl of IP buffer, eluted by boiling (95°C, 5 min) in sample buffer and analysed by immunoblotting. HA-NONO complexes were washed twice with 800 μl of IP buffer, split and eluted either as above or by heating (65°C, 5 min) in 2× urea loading dye (7 M urea, 0.05% xylene cyanol, 0.05% bromophenol blue) for separation by urea-PAGE (30 min, 350 V) in 1× TBE buffer (90 mM Tris, 90 mM boric acid, 2 mM EDTA), transfer on Whatman paper with a gel-dryer (BioRad) and detection by autoradiography with hyperfilms (Cytiva).

### BLISS-sequencing

Cells were washed in PBS, fixed (10 min at room temperature) with 5% paraformaldehyde, washed twice with PBS, lysed (1 h, 4°C) in lysis buffer 1 (10 mM Tris–HCl pH 8.0, 10 mM NaCl, 1 mM EDTA, 0.2% Triton X-100), washed in PBS, lysed again (1 h, 37°C) in lysis buffer 2 (10 mM Tris–HCl pH 8.0, 150 mM NaCl, 1 mM EDTA, 0.3% SDS) and washed twice in PBS. For AsiSI digestion, samples were twice equilibrated (2 min at room temperature) in 150 μl of 1× CutSmart buffer (NEB). A 3 μl aliquot of recombinant AsiSI endonuclease (10 U/μl, NEB) was added to replicates and incubated (2 h at 37°C). Controls were incubated in buffer only. For DSB blunting, samples were washed three times (2 min at room temperature) in 1× CutSmart buffer and incubated (1 h at room temperature) in 150 μl of blunting mix [112.5 μl of ddH_2_O, 15 μl of 10× blunting buffer (NEB), 15 μl of 100 μM dNTPs, 0.3 μl of 50 mg/ml bovine serum albumin (BSA), 6 μl of blunting enzyme mix from the quick blunting kit (NEB)]. Prior to ligation, 10 μM of corresponding BLISS (breaks labelling *in situ* and sequencing) adapters (Supplementary Table S5) were mixed in equimolar amounts and annealed (5 min at 95°C with gradient cooling to 25°C). For ligation, samples were washed three times (2 min at room temperature) in 1× CutSmart buffer, pre-incubated (5 min at room temperature) in 1× T4 ligase buffer (NEB), and incubated (18 h at 16°C with gentle shaking) in 150 μl of ligation buffer [124.5 μl of ddH_2_O, 15 μl of 10× T4 ligase buffer, 3 μl of 50 mg/ml BSA, 1.5 μl of 2000 U/μl T4 ligase (NEB), 6 μl of BLISS adapter pairs]. For removal of excess adapters, samples were incubated (1 h at 37°C, with gentle shaking) in high salt wash buffer (10 mM Tris–HCl pH 8.0, 2 M NaCl, 2 mM EDTA, 0.5% Triton X-100) and washed in PBS. For extraction of genomic DNA, samples were incubated (5 min at room temperature) in 100 μl of extraction buffer [10 mM Tris–HCl pH 8.0, 100 mM NaCl, 50 mM EDTA, 1% SDS, 10% 10 mg/ml proteinase K (Sigma)], harvested by scraping, pooled (merged conditions for each replicate) and incubated (18 h at 55°C). DNA was purified by phenol/chloroform extraction, recovered in 50 μl of ddH_2_O and sonicated (Covaris). Fragmented DNA was concentrated using SPRI select beads (Beckman) and a magnet (Alpaqua), washed twice with 80% ethanol, air-dried and eluted in 8 μl of ddH_2_O. For *in vitro* transcription (IVT), 7.5 μl of DNA was incubated (14 h at 37°C) with IVT mix [0.5 μl of Ribolock inhibitor (Invitrogen), 2 μl of T7 polymerase buffer (NEB), 8 μl of rNTP mix, 2 μl of T7 polymerase (Invitrogen)]. DNA was removed by addition of 1 μl of turbo DNase (Invitrogen) for 15 min. RNA size selection and clean-up was performed with RNAClean XP beads (Beckman); size-selected RNA was washed with 80% ethanol, air-dried and eluted in 6 μl of ddH_2_O. For library preparation, 1 μl of 5 μM RA3 adapter (NEB) was added to 5 μl of RNA sample, incubated (2 min at 70°C) and placed on ice. A 4 μl aliquot of ligation mix [2 μl of 10× T4 ligase buffer (NEB), 1 μl of T4 RNA ligase 2, truncated (NEB), 1 μl of Ribolock inhibitor] was added and incubated (1 h at 28°C). For reverse transcription (RT), 3.5 μl of ddH_2_O and 1 μl of 10 μM RTP primer (NEB) was added, incubated (2 min at 70°C) and placed on ice. A 5.5 μl aliquot of RT mix [2 μl of 5× GC buffer (Invitrogen), 0.5 μl of 12.5 mM dNTP mix, 1 μl of 100 mM dithiothreitol (DTT), 1 μl of SuperScriptIII reverse transcriptase (Invitrogen), 1 μl of Ribolock inhibitor] was added and incubated (1 h at 50°C) and heat inactivated (15 min at 70°C). For indexing and amplification, 10 μl of RT reaction was mixed with 25 μl of NEBNext 2× PCR mix, 2 μl of 10 μM RPI primer (NEB), 2 μl of 10 μM RP1 primer (NEB) and 1 μl of ddH_2_O, and PCR amplified for 16–18 cycles. Library clean-up was performed with AMPure XP beads (Beckman). The library was captured, washed twice with 80% ethanol, air-dried and eluted in 20 μl of ddH_2_O prior to sequencing.

### Chromatin immunoprecipitation (ChIP) and CUT&RUN-sequencing

For ChIP, cells were fixed with 1% formaldehyde (10 min at 37°C), quenched in 0.125 M glycine (10 min at 37°C), washed in PBS and centrifuged (2000 rpm, 5 min). Pellets were resuspended in 500 μl of cold cell lysis buffer (5 mM PIPES pH 8.0, 85 mM KCl, 0.5% NP-40, 1× protease/phosphatase inhibitor) and lysed (10 min on ice). Nuclei were centrifuged (3000 rpm, 5 min) and resuspended in 300 μl of cold nuclear lysis buffer (1% SDS, 10 mM EDTA, 50 mM Tris–HCl pH 8.0, 1× protease/phosphatase inhibitor) and lysed (10 min on ice). Lysates were sonicated (five times for 5 min, 30 s on/off) with a Bioruptor (Diagenode) and pelleted (13 000 rpm, 10 min). The supernatant was mixed with 2 ml of dilution buffer (0.01% SDS, 1.1% Triton X-100, 1.2 mM EDTA, 16.7 mM Tris–HCl pH 8.0, 167 mM NaCl, 1× protease/phosphatase inhibitor). Diluted samples were aliquoted and 5 μg of antibodies were added (IP sample) or not (input) and incubated overnight (4°C with rotation). For pull-down, 20 μl of Protein G Dynabeads were added to IP samples, incubated (1.5 h with rotation), immobilized on a magnet and washed with wash buffer A (0.1% SDS, 1% Triton X-100, 2 mM EDTA, 20 mM Tris–HCl pH 8.0, 150 mM NaCl), B (0.1% SDS, 1% Triton X-100, 2 mM EDTA, 20 mM Tris–HCl pH 8.0, 500 mM NaCl), C (0.25 M LiCl, 1% NP-40, 1% sodium deoxycholate, 1 mM EDTA and 10 mM Tris–HCl pH 8.0), and twice with D (10 mM Tris–HCl pH 8.0, 1 mM EDTA). For elution, samples were incubated with 500 μl of elution buffer (1% SDS, 0.1 M NaHCO_3_) for 30 min with rotation. Reversal of cross-links was performed at 65°C overnight after adding 30 μl of 5 M NaCl, 1 μl of 10 mg/ml RNase A (Sigma), 10 μl of 0.5 M EDTA, 20 μl of 1 M Tris–HCl pH 6.8, 2 μl of 10 mg/ml proteinase K (Sigma) to input and IP samples. DNA was purified by phenol/chloroform extraction, recovered in ddH_2_O and assessed by quantitative polymerase chain reaction (qPCR) with selective primers (Supplementary Table S6). For DNA–RNA hybrid IP (DRIP), non-cross-linked lysates were incubated (1 h at 37°C) with 10 U of RNase H (NEB) prior to immunoselection. For CUT&RUN-seq, unperturbed cells, or cells transfected with ppyCAG-V5-RNase H1 wild type or D210N mutant, were harvested with accutase (Sigma), centrifuged (2500 rpm, 3 min) and washed three times in 1.5 ml of wash buffer (20 mM HEPES pH 7.5, 150 mM NaCl, 0.5 mM spermidine). Cells were incubated (10 min at room temperature) with 10 μl of concanavalin A-coated magnetic beads (BioMag) resuspended in an equal volume of binding buffer (20 mM HEPES pH 7.5, 10 mM KCl, 1 mM CaCl_2_, 1 mM MnCl_2_), immobilized on a magnet, permeabilized with 150 μl of antibody buffer (20 mM HEPES pH 7.5, 150 mM NaCl, 0.5 mM spermidine, 0.05% digitonin, 2 mM EDTA) and incubated with 1 μg of primary antibody (800 rpm, 4°C, overnight with rotation). Samples were placed on a magnet, washed twice with 1 ml of DIG-wash buffer (20 mM HEPES pH7.5, 150 mM NaCl, 0.5 mM spermidine, 0.05% digitonin) and incubated (1 h, 800 rpm, 4°C with rotation) with 150 μl of protein A/G–micrococcal nuclease (MNase) fusion protein (1 μg/ml, CST). Reactions were placed on a magnet, washed with 1 ml of DIG-wash buffer twice and once with 1 ml of rinse buffer (20 mM HEPES pH 7.5, 0.05% digitonin, 0.5 mM spermidine). For chromatin digestion and release, samples were incubated (30 min, on ice) in ice-cold digestion buffer (3.5 mM HEPES pH 7.5, 10 mM CaCl_2_, 0.05% digitonin). The reaction was stopped by addition of 200 μl of stop buffer (170 mM NaCl, 20 mM EGTA, 0.05% digitonin, 50 μg/ml RNase A, 25 μg/ml glycogen) and fragments were released by incubation (30 min, 37°C). The supernatant was incubated (1 h at 50°C) with 2 μl of 10% SDS and 5 μl of proteinase K (10 mg/ml, Sigma). Chromatin was recovered by phenol/chloroform extraction and resuspended in 30 μl of TE (1 mM Tris–HCl pH 8.0, 0.1 mM EDTA). For sequencing, replicates were quantified with a fragment analyser (Agilent) and subjected to library preparation. Libraries for small DNA fragments (25–75 bp) were prepared based on the NEBNext Ultra II DNA library prep Kit for Illumina (NEB#E7645).

### RNA analytics

Total RNA was isolated using TRIzol (Invitrogen) following the manufacturer's protocol. cDNA was synthesized using SuperScriptIII reverse transcriptase (Invitrogen) with gene-specific primers (Supplementary Table S6) and quantified upon reverse transcription–quantitative PCR (RT–qPCR) in a thermocycler (Applied) with PowerUp SYBR green master mix (Applied) following the manufacturer's protocols. For dot blots, total RNA was extracted by TRIzol, resuspended in ddH_2_O with 0.02% methylene blue, heated (5 min, 72°C), spotted on a nylon membrane (Cytiva), cross-linked (120 mJ/cm^2^) using a UV-cross-linker (Analytic Jena), blocked in PBS/0.1% Triton X-100/0.5% SDS (20 min), washed with PBS/0.1% Triton X-100 (20 min), incubated (4°C, overnight) with a streptavidin–horseradish peroxidase (HRP) probe (Invitrogen), washed with PBS/0.1% Triton X-100 (20 min) and visualized with an ECL kit (Cytiva). For SYBR gold (Invitrogen) staining, immunoselected transcripts were on-bead digested (10 min at room temperature) with 2 μl of 10 μg/ml RNase A (Sigma), separated by urea-PAGE, stained with 1× SYBR gold diluted in 1× TBE (10 min in the dark) and visualized on a transilluminator (Thermo). For northern blot hybridization, 15 μg of total RNA was resuspended in 2× RNA loading dye (50% formamide, 15% formaldehyde, 1× MOPS buffer, 0.1% bromophenol blue, 10 μg/ml ethidium bromide) and separated on a 1.2% agarose gel containing 5.5% para-formaldehyde and 1× MOPS buffer (40 mM MOPS, 10 mM NaAc, 1 mM EDTA pH 7.0) for 90 min at 100 V. Separated RNA was transferred on a positively charged nylon membrane (Hybond N+, GE Healthcare) by semi-dry blotting overnight using 10× SSC buffer (1.5 M NaCl, 150 mM NaCitrate, pH 7.0), washed in ddH_2_O and UV-cross-linked using a UV-cross-linker (Analyik Jena, 120 mJ/cm^2^). Cross-linked RNA was pre-hybridized in ULTRAhyb Ultrasensitive hybridization buffer (Invitrogen) (4 h, 42°C) in a hybridization oven (UVP). For detection of cross-linked RNA, 1 μM DNA oligonucleotide probe (Supplementary Table S7) complementary to CDKN1A mRNA was end-labelled with 10 μl of T4 PNK-containing labelling mix [1 μl of 10× PNK buffer (NEB), 1 μl of 1 μM DNA probe, 1 μl of T4 PNK (NEB), 1 μl of [γ-^32^P]ATP (Hartmann), 6 μl of ddH_2_O] for 40 min at 37°C with rotation. The end-labelled probe was diluted in 40 μl of TE buffer (10 mM Tris, 1 mM EDTA), purified by centrifugation (5 min, 3200 rpm) using pre-equilibrated Microspin G-25 columns (Invitrogen), boiled (95°C, 5 min), added to hybridization tubes containing pre-hybridized membranes, and incubated (36 h, 42°C). Membranes were washed twice in 1× SSC buffer (15 mM sodium citrate, 150 mM NaCl) for 10 min at 42°C with rotation, air-dried and subjected to autoradiography. Signals were quantified using ImageJ (NIH). For control of loading and size, rRNA was visualized by ethidium bromide staining under UV light.

### mNET-IP and mNET-sequencing

For mNET-IP (mammalian nascent elongation transcript-IP), 5 μg of antibodies were coupled to Protein G Dynabeads (Invitrogen), washed and resuspended in 100 μl of NET-2 buffer (50 mM Tris–HCl pH 7.4, 150 mM NaCl, 0.05% NP-40) prior to immunoselection. Cells were harvested, washed in PBS and lysed in hypotonic lysis buffer (10 mM HEPES pH7.9, 60 mM KCl, 1.5 mM MgCl_2_, 1 mM EDTA, 1 mM DTT, 0.075% NP-40, 400 U of Ribolock inhibitor, 1× protease/phosphatase inhibitor) (10 min, 4°C with rotation). Nuclei were centrifuged (2 min, 1000 rpm), washed twice in hypotonic lysis buffer without NP-40 and resuspended in 125 μl of cold NUN1 buffer (20 mM Tris–HCl pH 7.9, 75 mM NaCl, 0.5 mM EDTA, 50% glycerol, 400 U of Ribolock inhibitor, 1× protease/phosphatase inhibitor). A 1.2 ml aliquot of NUN2 buffer (20 mM HEPES-KOH pH 7.6, 300 mM NaCl, 0.2 mM EDTA, 7.5 mM MgCl_2_, 1% NP-40, 1 M urea, 400 U of Ribolock inhibitor, 1× protease/phosphatase inhibitor) was added and nuclei were incubated on ice (15 min, sporadic vortexing) and centrifuged (10 min, 16 000 rpm). The non-soluble chromatin pellet was washed in 100 μl of 1× MNase buffer (NEB), centrifuged and digested (2 min, 37°C with rotation) in 100 μl of MNase reaction mix [87 μl of ddH_2_O, 10 μl of 10× MNase buffer (NEB) 1 μl of 100× BSA, 2 μl of 2000 U/μl MNase (NEB)]. MNase digests were centrifuged (5 min, 16 000 rpm) and the supernatant was diluted with 10 vols of NET-2 buffer. Prior to dilution, 10% of MNase digests (input) were taken. Conjugated antibodies were added to the diluted supernatants and incubated (2 h, 4°C with rotation). Immunocomplexes were immobilized on a magnet (Invitrogen) and washed three times in 800 μl of NET-2 buffer. For analysis of proteins, input and mNET-IP samples were analysed by immunoblotting as above. For analysis of immunoselected transcripts, 10% of the mNET-IP sample was subjected to TRIzol extraction and RT-qPCR or end-labelled on beads with radioactive PNK labelling mix and analysed by autoradiography or monitored for enrichment by immunoblotting. Then 90% of the mNET-IP sample was end-labelled on beads with non-radioactive PNK labelling mix, eluted and separated by urea-PAGE along with inputs as above. A small RNA (<100 nt) fraction was size-selected according to methylene blue migration. Slices were incubated (2 h at room temperature with rotation) in 400 μl of elution buffer (1 M NaOAc, 1 mM EDTA) and centrifuged (2 min, 13 000 rpm). Supernatants containing eluted RNA were loaded on spin-x-columns (Coster) and centrifuged (1 min, 13 000 rpm). Flow-through was precipitated using 1 ml of 100% ethanol and 1 μl of glycogen (Invitrogen), incubated (20 min at room temperature) and centrifuged (20 min, 13 000 rpm). Pellets were washed in 70% ethanol, air-dried and recovered in 6 μl of ddH_2_O. RNA quality was controlled using a fragment analyzer (Agilent). For mNET-seq, three biological replicates were pooled and subjected to library preparation. Libraries were prepared using a NEBNext Multiplex small RNA library prep Kit (NEB#7300) following the manufacturer's protocol.

### 4sU-tagging, APEX2-mediated proximity ligation and RNA sequencing

For 4sU-tagging, cells were incubated with 4sU (Sigma, 2 mM) for 15 min, directly lysed in 2.1 ml of QIAzol (Qiagen), spiked with 4sU-labelled mouse cell lysates, and total RNA was extracted using an miRNeasy kit (Qiagen) following the manufacturer's protocol. A 50 μg aliquot of total RNA was diluted in 100 μl of ddH_2_O, denaturated (5 min, 65°C), put on ice (10 min) and incubated (2 h at room temperature) with 50 μl of biotin-HPDP (Thermo, 1.85 mM) diluted in 100 μl of 2.5× biotin labelling buffer (25 mM Tris–HCl pH 7.4, 2.5 mM EDTA). The reaction was mixed with an equal volume of chloroform/isoamyl alcohol (24:1) and separated with a phase-lock tube (Qiagen) by centrifugation (12 000 rpm, 5 min). RNA was precipitated (5 min at room temperature) with 1 μl of glycogen (Invitrogen), 20 μl of 5 M NaCl and an equal volume of isopropanol, and centrifuged (14 000 rpm, 20 min, 4°C). The pellet was washed in 500 μl of 75% ethanol, centrifuged (14 000 rpm, 10 min, 4°C) and resuspended in 100 μl of ddH_2_O. For APEX2-mediated proximity labelling, cells were incubated (30 min, 37°C) with 0.5 mM biotin–phenol (Iris), pulsed (1 min) with 1 mM H_2_O_2_ (Sigma) and quenched by 10 mM sodium ascorbate (Sigma), 5 mM trolox (Sigma) and 10 mM sodium azide (Sigma). Cells were directly lysed in 2.1 ml of QIAzol (Qiagen), and total RNA was extracted using an miRNeasy kit (Qiagen) following the manufacturer's protocol. For selection of biotinylated transcripts, samples were incubated (15 min at room temperature) with 50 μl of streptavidin T1 Dynabeads (Thermo) and resuspended in 100 μl of 2× washing buffer (2 M NaCl, 10 mM Tris–HCl pH 7.5, 1 mM EDTA, 0.1% Tween-20). Immunocomplexes were immobilized on a magnet and washed three times with 1× washing buffer (1 M NaCl, 5 mM Tris–HCl pH 7.5, 0.5 mM EDTA, 0.05% Tween-20). For elution, samples were incubated with 100 μl of DTT (100 mM) at room temperature for 5 min and recovered using an RNeasy MinElute clean up kit (Qiagen) following the manufacturer's protocol. For APEX-seq, biotinylated RNA was enriched by incubation with 20 μl of streptavidin C1 Dynabeads (2 h, 4°C) pre-washed three times with washing buffer (5 mM Tris–HCl, pH 7.5, 0.5 mM EDTA, 1 M NaCl, 0.1% Tween-20), twice with solution A (100 mM NaOH, 50 mM NaCl) and resuspended with solution B (100 mM NaCl). Immunocomplexes were immobilized on a magnet, washed three times with washing buffer and resuspended in 54 μl of ddH_2_O. For elution, samples were incubated (1 h at 42°C followed by 1 h at 55°C with shaking) with 33 μl of 3× proteinase digestion buffer [330 μl of 10× PBS, 330 μl of 20% *N*-laurylsarcosine sodium solution (Sigma Aldrich), 66 μl of 0.5 M EDTA, 16.5 μl of 1 M DTT, 357.5 μl of ddH_2_O], 10 μl of proteinase K (20 mg/ml) and 3 μl of Ribolock inhibitor, and recovered using an RNA clean and concentrator kit (Zymo) following the manufacturer’s protocol. For 4sU-sequencing and APEX-seq, three biological replicates were quantified by RiboGreen assay (Thermo) following the manufacturer's protocol, pooled and subjected to library preparation. Libraries were prepared using an NEBNext Ultra II Directional RNA library prep Kit (NEB#E7760) and an NEBNext rRNA Depletion Kit (NEB#E6310) following the manufacturer's protocol.

### Enhanced cross-linking immunoprecipitation and sequencing (eCLIP-sequencing)

Cells cultured in the absence or presence of etoposide were washed with PBS, subjected to UV irradiation (200 mJ/cm^2^), scraped, resuspended in ice-cold PBS, pelleted (1200 rpm, 5  min) and stored at −80°C. The pellets were lysed (20 min, 4°C) in eCLIP lysis buffer (50 mM Tris–HCl pH 7.4, 150 mM NaCl, 1 mM EDTA, 1% NP-40, 0.5% sodium deoxycholate, 0.25 mM TCEP). Subsequently, a limited RNase I (Invitrogen) and TURBO DNase (Invitrogen) digest (20 min, 37°C) was performed. For IP, the NONO antibody (5 μg/mg total protein) was coupled to 30 μl of Protein G Dynabeads (Invitrogen) and incubated with lysates (4°C, overnight). The samples were washed twice in eCLIP lysis buffer, twice in wash buffer (50 mM Tris–HCl pH 7.4, 300 mM NaCl, 1 mM EDTA, 1% NP-40, 0.5% sodium deoxycholate, 0.25 mM TCEP), followed by two washes in low-salt wash buffer (50 mM Tris–HCl pH 7.4, 1 mM EDTA, 0.5% NP-40). Subsequent library preparation was performed as described (26).

### Imaging

Cells were grown on cover slips (Roth), washed in PBS, fixed (10 min) in 3% paraformaldehyde (Sigma), washed in PBS (three times, 5 min), permeabilized with PBS/0.1% Triton X-100 (10 min) and blocked with PBS/10% FBS (2 h, 4°C). Primary and secondary antibodies (Supplementary Table S3) were diluted in PBS/0.15% FBS and incubated in a humidified chamber (overnight at 4°C or 2 h at room temperature), respectively. Cells were washed between incubations with PBS/0.1% Triton X-100 (three times for 5 min), sealed in 4′,6-diamidino-2-phenylindole (DAPI)-containing mounting medium (Vectashield), and imaged by confocal microscopy (CLSM-Leica-SP2 or -SP8, 1024 × 1024 resolution, ×63, airy = 1). Channels were acquired sequentially, between frames, with equal exposure times. More than 100 cells per condition were quantified. Pan-nuclear localization was scored in cells that display a homogenous nuclear staining and co-localization with nucleolar markers, which was assessed by using RGB profiler (ImageJ) and by the calculation of the Pearson's correlation coefficient using JACoP (ImageJ). Proximity ligation assays (PLAs) were performed with a Duolink in-situ PLA kit (Sigma) following the manufacturer's protocol. RNA-FISH (fluorescence *in situ* hybridization) experiments were performed with a panel of 30 non-overlapping, Quasar570-labelled sense DNA probes reverse complementary to 0.6 kb of the mapped region of antisense transcription at IGS-22 (Stellaris probe designer, masking level ≥ 2, Biosearch, Supplementary Table S8) following the manufacturer's protocol. Data were analysed using ImageJ (NIH).

For imaging upon RNP transfections, cells were grown on glass coverslips, fixed 8 h after RNP transfection using 4% paraformaldehyde (12 min at room temperature) and permeabilized with 0.5% Triton X-100 (10 min at room temperature). Samples were incubated at room temperature with primary antibody for 60 min, with secondary antibody for 30 min and with 5 mg/ml DAPI (Invitrogen) for 5 min in a moist chamber. Cells were washed three times in PBS between stainings, rinsed with water, mounted with Vectashield Mounting medium (Vector Laboratories) and sealed with nail polish. Qualitative image analysis of fluorescence was carried out using a point scanning confocal microscope LSM800 (Zeiss) with the Plan-Apochromat ×40 oil immersion objective and ZEN Software (Zeiss). The scoring of pan-nuclear NONO or nucleolar integrity using nucleophosmin 1 (NPM1) antibody was performed manually. A minimum of 135 cells were scored per condition. The experiment was performed three times.

### Generation of FASTQ, BAM and bedgraph files

For APEX-seq, CUT&RUN-seq and 4sU-seq, base calling was performed using Illumina's FASTQ Generation software v1.0.0, and sequencing quality was tested using FastQC. Reads were mapped with STAR v2.7.10a (4sU-seq) (27) or Bowtie2 v2.3.5.1 (28) (other experiments) to human hg19, human T2T, mouse mm10 or the *Escherichia coli* genome. Mouse reads were used for spike-normalization based on a scaling factor calculated for each 4sU-sequencing dataset as described (29). CUT&RUN-seq read normalization was performed by the sample-wise division of hg19-mapped reads by *E. coli*-mapped reads. This ratio was then multiplied by the smallest number of *E. coli*-mapped reads for each sample. For 4sU-seq, only the reads falling in introns were considered, BAM files obtained after spike normalization were sorted and indexed using SAMtools v1.9, and Bedgraph files were generated using the genomecov function from BEDTools v2.26.0 (30). The Integrated Genome Browser was used to visualize these density files.

### Generation of density and volcano plots

For CUT&RUN-seq, density plots of the indicated gene group were generated with ngs.plot using normalized bam files, testing the 1000 most expressed genes in U2OS (31). Then 1% extreme values were trimmed by using the option ‘–RB 0.01’. For APEX-seq, gene expression was assessed with featureCounts v2.0.3 (32) using bam files with intron-containing, non-spliced reads. Differential gene expression was assessed with edgeR v3.24.1 (Galaxy) (33) using Benjamini–Hochberg-adjusted *P*-value < 0.05, an expression filter rejecting genes with < 100 counts, and excluding non- and weakly expressed genes. For 4sU-seq, counts that overlap between the spike-normalized bam files and human genes (GRCh37.p13) were assessed with bedtool Intersect intervals v2.29.2 (Galaxy) (33), testing the 1000 most expressed genes in U2OS (31). The read count mapped reads for each condition were used to generate scatter plots with GraphPad.

### Bioinformatic analysis of mNET-seq

For mapping of mNET-seq data to rDNA loci, the FASTQ files were aligned to a custom reference genome based on the human rDNA complete repeating unit, GenBank: U13369.1. Alignment was performed using Bowtie2 allowing one mismatch, and aligned reads were depth normalized to have the same number of aligned reads as the sample with the least number of aligned reads. For visualization over the Integrated Genome Browser, the aligned reads were converted into bedGraphs carrying equal number of reads in the rDNA region and shown along with annotations based on U13369.1 and custom entries. For analysis of IGS loci, paired-end samples were mapped to human genome CHM13 (version 1.1) using Bowtie2 with the pre-set parameter ‘very-sensitive-local’, and normalized relative to spiked-in reads mapping to the mouse genome GRCm38/mm10. A bed file with the coordinates of the 214 rDNA-IGS (derived from 219 rDNA loci, arranged in five clusters) was extracted from the gff3-file for CHM13 draft annotation version 1.1, and used to generate a multifasta file, containing a total of 6.8 million nucleotides. The sequences of 470 Alu repeats (representing 50 different subfamilies) were derived from the human genome version GRCh38/hg38, using the coordinates from the rmsk-table at UCSC, for a total length of 112 000 nucleotides. IGS sequences not matching any of these Alu elements were identified with blastn, resulting in 6.3 million nucleotides of ‘non-Alu’ IGS which were subsequently divided into bins of 100 nt each. The number of spike-normalized reads mapping to each bin was determined using bedtools intersect, and numbers for bins overlapping a feature of interest (a PCR probe or an individual IGS) were pooled.

### Bioinformatic analysis of BLISS-seq

Analysis was performed as described (34). Samples were demultiplexed based on their condition-specific barcodes using UMI-tools, allowing one mismatch in the barcode, and separately mapped to hg19 using Bowtie2 with default parameters. Three biological triplicates were merged prior to mapping, and collectively processed. Samples were filtered against an ENCODE Blacklist file to remove regions of high variance in mappability using bedtools intersect. To allow absolute quantification of DSBs and remove PCR-introduced artefacts, duplicated reads were identified based on their UMI, grouped and deduplicated using UMI-tools with default parameters. Density profiles were generated using the R package metagene2 with the assay parameter ‘ChIPseq’ with 200 bp read extension. The bar graph was generated using the R package exomeCopy in the respective regions up- and downstream of the annotated transcription start site (TSS) and divided by the number of genes in the corresponding gene set. Publicly available RNA-sequencing data (ENCODE: ENCFF182XEY) were filtered using gene length (≥ 1500 bp) and subsequently used to stratify genes by expression into genes that were highly expressed [fragments per kilobase of transcript per million mapped reads (FPKM) > 10] and those which were expressed at a low level (FPKM ≤ 1). Promoters with proximal downstream TSSs were removed.

### Bioinformatic analysis of eCLIP-seq

For bioinformatic analysis of eCLIP-seq data, paired-end sequencing reads from eCLIP experiments were initially trimmed using a custom Python script to identify the unique molecular identifier (UMI). These trimmed reads were then aligned to the human genome (hg38, ensemble v100) using the Burrows–Wheeler Aligner (BWA, 0.7.17-r1188). Next, PCR duplicates were removed using Picard's MarkDuplicates (v2.23.3–1) with UMI-aware deduplication. Finally, enriched protein-binding regions were identified by MACS2 callpeak (v.2.2.7) with parameters ‘-g hs -s 58 -B –keep-dup all –nomodel –extsize 50 –d-min 5 –scale-to small –B’, comparing IP and size-matched INPUT samples. Visualizations of the region were rendered from the PCR-deduplicated .bam files using the Integrated Genome Browser. Distribution analysis employed the ChIPpeakAnno package and the TxDb.Hsapiens.UCSC.hg38.knownGene dataset, with plots generated using ggplot2.

## Results

### Etoposide treatment enriches NONO in the nucleolus

We showed previously that short-term incubation with etoposide (2 h, 20 μM) causes the accumulation of a recombinant NONO fusion protein in nucleoli that comprises a canonical, non-disintegrated tripartite structure (35). We initially confirmed this by co-staining of endogenous DBHS proteins NONO, SFPQ or PSPC1 with the nucleolar marker NPM1 (Supplementary Figure S1A). The etoposide-induced nucleolar re-localization of NONO was sensitive to pre-incubation with the ATM inhibitor KU-55933 and could not be observed upon incubation with the transcriptional kinase inhibitor THZ1. The latter caused prominent translocation of NPM1 to the nucleoplasm instead (Supplementary Figure S1B). As expected, etoposide treatment increased the DNA damage marker Ser139-phosphorylated histone H2.X variant (γH2A.X) >5-fold, but not DBHS proteins, whilst pre-incubation with ATM inhibitor impaired the etoposide-responsive onset of γH2A.X (Supplementary Figure S1C, D). To test if NONO re-localization is induced by locus-specific DSBs, we employed the 4-OHT-inducible endonucleases I-PpoI and AsiSI. Both enzymes cleave nucleoplasmic loci, but induce DSBs also in the nucleolar 28S rDNA and the nucleolar 5′ETS sequence, respectively (36,37). We observed signals for DSB markers γH2A.X and the p53-binding protein 1 (53BP1), but little nucleolar re-localization of NONO or co-localization with the DSB markers upon transient transfection of endonucleases and 4-OHT treatment (Supplementary Figure S2A, B). Furthermore, etoposide treatment induced prominent formation of DNA topoisomerase II-binding protein 1 (TOPBP1)-positive foci in the nucleoplasm, whilst I-PpoI or AsiSI cleavage caused TOPBP1 accumulation around nucleolar caps (Supplementary Figure S2C, D). To assess NONO localization upon selective induction of DSBs in the nucleolus or nucleoplasm, we used RNP transfection in U2OS cells. We transfected recombinant purified Cas9 endonuclease complexed with sgRNA targeting either nucleolar (5′ETS/IGS) or nucleoplasmic (5S) loci or non-targeting control sgRNA, and compared NONO localization upon co-staining with NPM1 (Supplementary Figure S2E). Strikingly, transfection of RNPs containing 5S-targeting sgRNA, but not nucleolar rDNA-targeting or control sgRNA increased the number of cells with pan-nuclear NONO localization ∼2-fold. To monitor for the selective induction of DSBs, we repeated RNP transfections and co-stained for γH2A.X and the Treacle ribosome biogenesis factor 1 (TCOF1). As expected, RNP transfection with sgRNAs that target Cas9 to the nucleolus induced prominent formation of TCOF1-positive nucleolar caps and γH2A.X foci in the periphery of disintegrated nucleoli, which was accompanied by NPM1 translocation, and is indicative for nucleolar disintegration (38) (Supplementary Figure S2F). In contrast, 5S-cleaving RNPs induced nucleoplasmic foci, but neither TCOF1-positive nucleolar caps nor NPM1 translocation. Moreoever, NPM1 translocation and the formation of TCOF1-positive nucleolar caps was only detectable upon prolonged incubation with a higher concentration of etoposide (4 h, 100 μM) (Supplementary Figure S2G, H). We conclude that a transient induction of DDR signalling upon etoposide treatment, probably induced by a heterogenous population of predominantly nucleoplasmic DSBs, is a prerequisite for the re-localization of NONO into non-disintegrated nucleoli.

### The RRM1 domain facilitates NONO nucleolar re-localization

**Figure 1.**
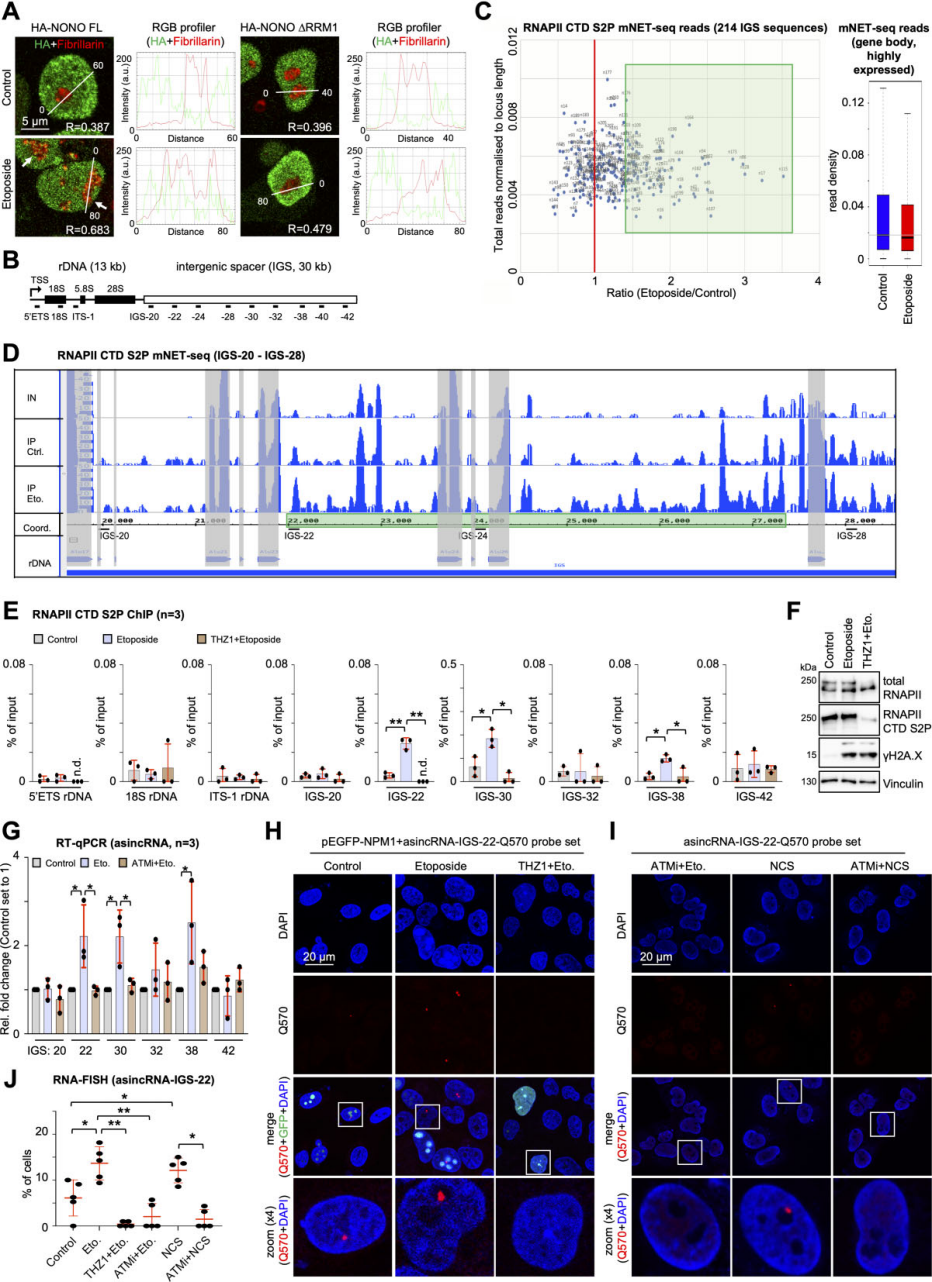
DNA damage induces RNAPII-dependent nucleolar transcripts in U2OS cells. (**A**) Imaging and quantitation (line scans) of HA-NONO variants and fibrillarin. Arrowhead, co-localization; R = Pearson correlation. (**B**) Scheme of human rDNA array (∼80 repeats on chromosomes 13, 14, 15, 21, 22). IGS 20–42, probe positions in kilobases downstream of the rDNA TSS. (**C**) Scatter plot displaying mNET-seq reads for the IGS (left) and the top 1000 expressed protein-coding genes (right). Green box, induced transcripts. (**D**) mNET-seq browser tracks for IGS consensus region 20–28 from inputs (IN, merged) or after IP with CTD S2P-selective antibody ± etoposide. Grey, Alu element; green, induced region. (**E**) CTD S2P ChIP with site-specific primers. (**F**) Immunoblots detecting total RNAPII, CTD S2P and γH2A.X. Vinculin = loading control. (**G**) RT–qPCR assessing transcript levels from total RNA ± etoposide or pre-treatment with ATM inhibitor. (**H**) Imaging of Quasar570 RNA-FISH signals originating at IGS-22 upon ectopic expression of GFP–NPM1. (**I**) as in (H) without ectopic expression of GFP–NPM1. White box, zoom. (**J**) Quantitation of (H) and (I). Each dot represents the percentage of cells with Quasar570-positive signals as the average from two acquisitions. More than 100 cells were assessed. *P-value < 0.05; **P-value < 0.001; two-tailed *t*-test; n.d., not detected. Error bar, mean ± SD. Representative images are shown. *n* = number of biological replicates.

RRM1/2 mediate the binding of NONO to nucleic acids (14). To test which domain confers nucleolar re-localization, we created HA-NONO mutants (Supplementary Figure S3A), monitored their expression (Supplementary Figure S3B) and assessed their localization (Figure 1A; Supplementary Figure S3C, D). Co-staining of mutants with fibrillarin revealed that full-length (FL) HA-NONO, the RRM1 deletion mutant (ΔRRM1) and the C-terminal deletion mutant (ΔC-ter) localized in the nucleoplasm in unperturbed cells. Upon incubation with etoposide, FL and ΔC-ter, but not ΔRRM1, displayed pan-nuclear localization and co-staining with fibrillarin. Thus, RRM1 confers nucleolar re-localization of NONO.

### RNAPII produces DNA damage-induced nucleolar transcripts at distinct IGS loci

Non-ribosomal, nucleolar transcripts maintain homeostasis by sequestration of RBPs (39,40). Intriguingly, C-terminal domain Ser2-phosphorylated RNAPII (CTD S2P) synthesizes antisense intergenic non-coding RNA (asincRNA) on nucleolar chromatin to regulate RNA polymerase I (RNAPI) in unperturbed cells (41). We hypothesized that the DDR modulates nucleolar RNAPII activity and applied mNET-seq to profile RNAPII-associated transcripts. First, we confirmed enrichment of RNAPII and associated transcripts upon immunoselection (Supplementary Figure S4A). CTD S2P mNET-seq revealed that etoposide treatment elevated ∼25% of the 214 individually mapped IGS sequence reads 2- to 3-fold, but not protein-coding sequences (Figure 1B, C). Pair-wise comparison further suggested a locus-specific increase in asincRNA at IGS loci 22, 30 and 38 (Supplementary Figure S4B), which was also visualized on browser tracks (Figure 1D; Supplementary Figure S4C–F). Next, we used ChIP to assess CTD S2P occupancy. Treatment with etoposide, but not pre-incubation with THZ1, elevated CTD S2P signals at IGS loci 22, 30 and 38 (Figure 1E). Importantly, etoposide had little impact on CTD S2P marks (Figure 1F). We confirmed the increase of asincRNA levels upon etoposide treatment as well as their sensitivity to ATM inhibition and THZ1 treatment by RT–qPCR from total RNA and from transcripts associated with CTD S2P, respectively (Figure 1G; Supplementary Figure S4G). In contrast, the levels of sense-oriented sincRNA were sensitive to etoposide and partially rescued by pre-incubation with ATM inhibitor (Supplementary Figure S4H). We also induced DNA damage with the radiomimetic drug NCS and confirmed both elevated CTD S2P occupancy at the IGS and increased asincRNA levels in the presence of NCS (Supplementary Figure S4I–K). Furthermore, we employed U2OS DIvA cells, which stably express the 4-OHT-inducible endonuclease AsiSI, to induce locus-specific DSBs (42). Confocal imaging was used to validate the DIvA system and confirmed prominent co-localization of γH2A.X- and 53BP1-positive foci upon incubation of DIvA, but not wild-type U2OS cells with 4-OHT (Supplementary Figure S5A). Of note, few such foci were detectable even in the absence of 4-OHT in DIvA cells, pointing toward some leakiness of the system. When assessing asincRNA levels by RT–qPCR, we found no significant induction of asincRNA across the IGS upon induction of AsiSI cleavage and subsequent nucleolar disintegration (Supplementary Figure S5B). Lastly, we performed RNA-FISH to visualize prominently induced asincRNA-IGS-22 and observed an increased number of cells comprising nucleolar RNA-FISH signals upon etoposide or NCS treatment, which were sensitive to pre-incubations with THZ1 and ATM inhibitor, respectively (Figure 1H–J). We conclude that CTD S2P produces asincRNA.

### asincRNAs form IGS R-loops to promote NONO re-localization

**Figure 2.**
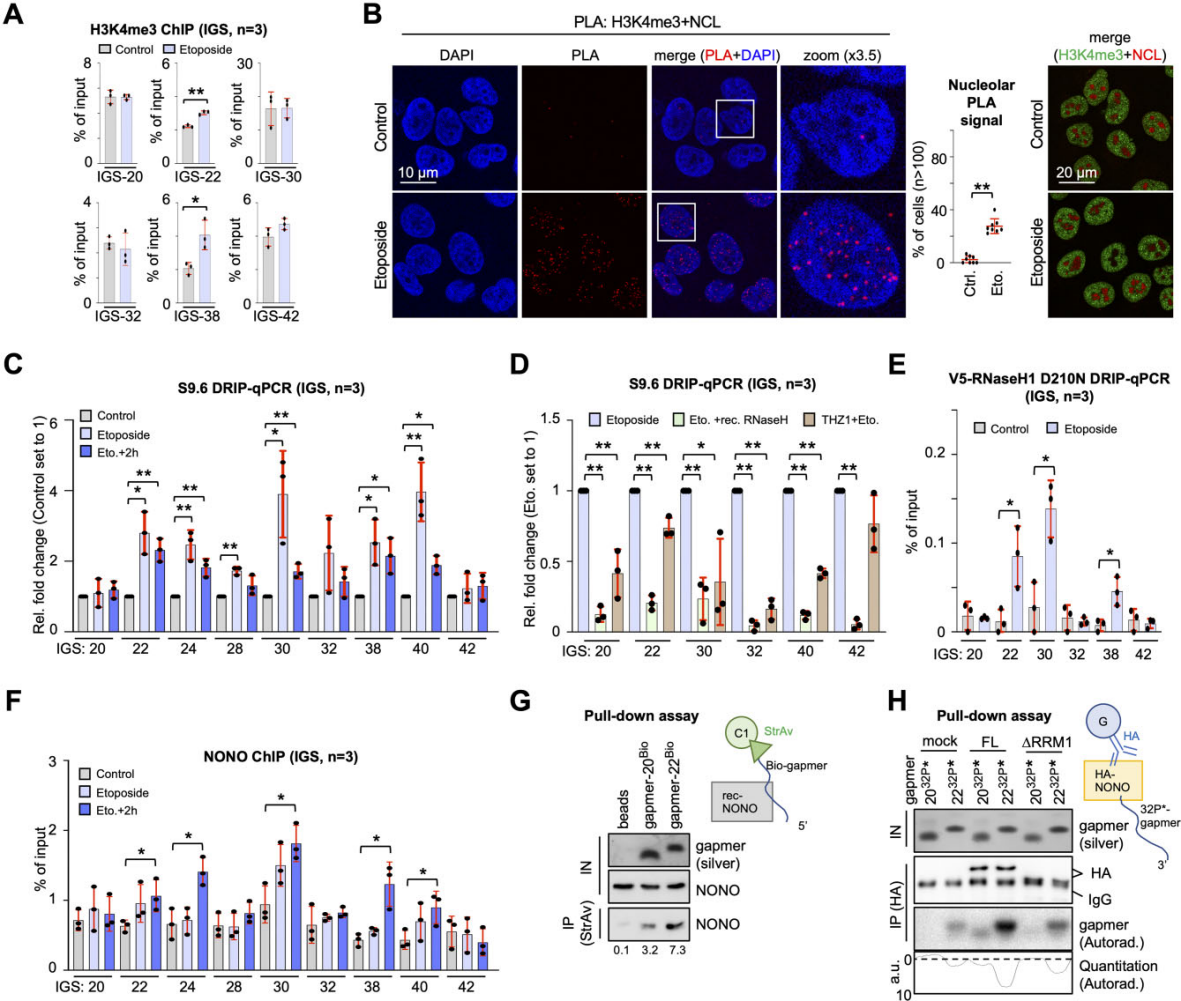
Elevated levels of H3K4me3 marks and R-loops correlate with NONO IGS occupancy in U2OS cells. (**A**) H3K4me3 ChIP using site-specific primers. (**B**) Imaging and quantitation of PLA signals (left) or indirect immunofluorescence signals (right) for H3K4me3/NCL. Each dot represents one acquisition. n, number of cells; white box, zoom. (**C** and **D**) Quantitative PCR of DNA immunopurified from DRIP-qPCR using S9.6 antibody and region-specific primers upon etoposide pulse–chase (C) or RNase H digestion/pre-treatment with THZ1 (D). (**E**) DRIP-qPCR using V5 antibody and region-specific primers after transient transfection with ppyCAG-V5-RNaseH1 D210N plasmid. (**F**) NONO ChIP using site-specific primers. (**G**) Pull-down assay displaying recombinant (rec)-NONO by immunoblotting after IP with biotin end-labelled (Bio) and immobilized gapmers. Silver stain and immunoblot of input (IN), loading controls; StrAv, streptavidin. (**H**) Pull-down assay displaying [γ-^32^P]ATP-end-labelled (32P*) gapmers by autoradiography after IP with immobilized HA-NONO variants FL and ΔRRM1, and PAGE separation. Silver stain and immunoblot, loading controls; dashed line, background; a.u., arbitrary units. *P-value < 0.05; **P-value < 0.001; two-tailed *t*-test. Error bar, mean ± SD. Representative images are shown. n = number of biological replicates.

The nucleolar chromatin has been mapped and comprises a prominent peak for a subset of histone modification marks within the IGS-30 region (43). Since asincRNA levels at IGS-22, 30 and 38 are responsive to DNA damage, we hypothesized that etoposide may alter histone marks within the IGS to regulate NONO re-localization. We selected two marks for open chromatin (histone H3 Lys4 trimethylation H3K4me3, and H3 Lys27 acetylation H3K27ac) and one for DNA damage (γH2A.X), and assessed their levels by ChIP. We found that etoposide treatment selectively increased the level of H3K4me3, but not H3K27ac or γH2A.X at nucleolar IGS-22 and 38 loci (Figure 2A; Supplementary Figure S6A, B). In contrast, γH2A.X levels were responsive to etoposide treatment at the promoter region of two protein-coding gene loci (Supplementary Figure S6C). To visualize the formation of open chromatin marks in close proximity to the nucleolus, we performed PLAs using selective antibodies for open chromatin marks and the nucleolar marker NCL. Indeed, etoposide treatment increased nucleolus-associated PLA signals upon co-staining for NCL with H3K4me3, but not H3K27ac (Figure 2B; Supplementary Figure S6D).

Nucleolar asincRNAs form R-loops to shield RNAPI from the IGS (41). As asincRNA-coding IGS loci and asincRNA-encoding regions overlap, we asked if IGS R-loops may mediate NONO nucleolar re-localization. We used S9.6 and NONO antibodies in DRIP and NONO ChIP experiments. For S9.6 validation, we employed U2OS DIvA cells. We assessed S9.6 reactivity at the R-loop-forming DS1 site (*RBMXL1* promoter) (42). We detected DRIP signals at DS1 in the presence of 4-OHT, which were modestly elevated compared with the non-induced control and sensitive to RNase H digestion (Supplementary Figure S6E). Next, we used S9.6 DRIP to determine R-loop levels on nucleolar chromatin (Figure 2C, D). We found that etoposide increased DRIP signals across the body of the IGS, which largely remained stable after 2 h of chase, but were sensitive to RNase H digestion or pre-incubation with THZ1. To corroborate our findings, we employed a V5 antibody and repeated the DRIP assay upon transient expression of V5-RNaseH1 D210N, a catalytically dead RNase H1 mutant that binds and stabilizes R-loops (Figure 2E). Again, we found elevated levels of R-loops at IGS loci 22, 30 and 38 in the presence of etoposide. To test if the formation of IGS R-loops correlates with elevated occupancy of NONO on nucleolar chromatin, we performed NONO ChIP assays. For validation of the NONO antibody, we first wished to assess NONO occupancy at DS1. NONO occupancy at DS1 was modestly reduced compared with the non-induced control and sensitive to NONO depletion by shRNA (Supplementary Figure S6F, G). Of note, the lentiviral transduction protocol resulted in a high number of cells in the G_1_ phase concomitant with a modest impairment of S-phase progression after NONO depletion (Supplementary Figure S6H). Next, we measured NONO occupancy on nucleolar chromatin. NONO ChIP signals were detectable on rDNA, but not responsive to etoposide (Supplementary Figure S6I). On the IGS, however, NONO occupancy was modestly increased upon etoposide treatment, in particular after 2 h of chase and at regions that displayed increased levels of asincRNA and R-loops (Figure 2F). The etoposide-induced accumulation of NONO at IGS loci 22, 30 and 38 was also sensitive to overexpression of GFP–RNase H1 (Supplementary Figure S6J). Importantly, neither etoposide treatment nor overexpression of GFP–RNase H1 or the combination thereof altered the cell cycle progression significantly (Supplementary Figure S6K). The overexpression of GFP–RNase H1 also did not induce DSB signalling *per se*, nor alter the strength of DSB signalling in the presence of etoposide (Supplementary Figure S6L). To assess if NONO binds R-loop-forming IGS sequences directly, we performed pull-down assays with recombinant NONO and end-labelled DNA–RNA chimeras (gapmers). Gapmers were designed with sequence complementary to asincRNA-encoding region IGS-22, or IGS-20 control, to mimic single-stranded (ss)DNA within R-loops. When incubating rec-NONO with immobilized biotinylated gapmers, we found that gapmer-22 enriched rec-NONO > 2-fold more strongly than gapmer-20 (Figure 2G). Next, we immobilized FL or ΔRRM1 HA-NONO variants on beads and incubated them with radiolabelled gapmers (Figure 2H; Supplementary Figure S6M). Immobilized FL, but not ΔRRM1, enriched gapmer-22 >2-fold more strongly than gapmer-20. Likewise, transient transfection of gapmer-22, but not gapmer-20, interfered with the subcellular localization of both NONO and NPM1 irrespective of etoposide treatment (Supplementary Figure S7A). Transfection of gapmer-22 triggered the formation of nucleoplasmic NONO puncta concomitant with elevated levels of γH2A.X and reduced viability (Supplementary Figure S7B, C). These data suggest that etoposide induces IGS R-loops at regions of open chromatin to promote NONO binding to ssDNA sequences present within a subset of IGS R-loops.

### DNA damage reduces NONO occupancy at protein-coding gene promoters and attenuates pre-mRNA synthesis

**Figure 3.**
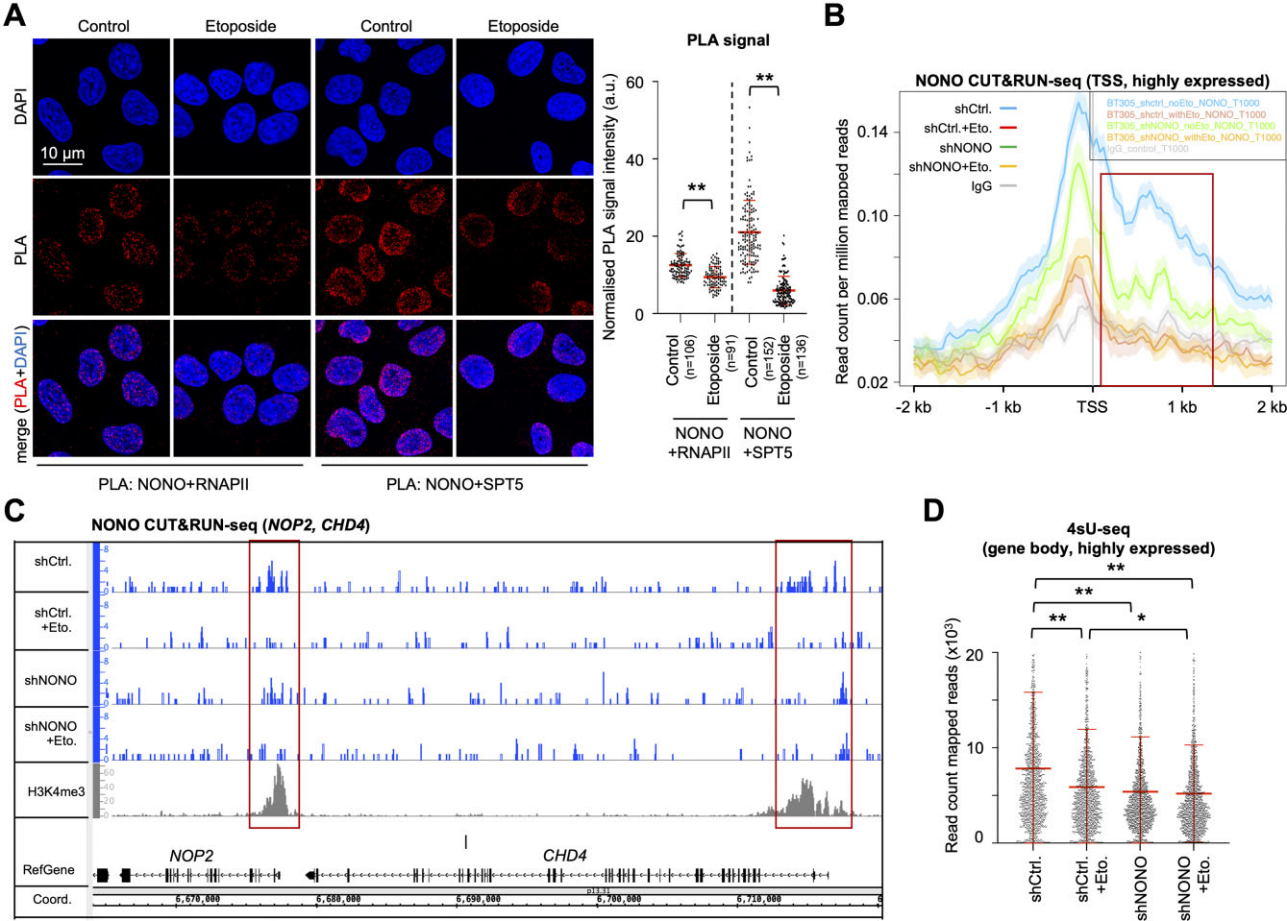
DNA damage reduces promoter-associated occupancy of NONO and RNAPII activity in U2OS cells. (**A**) Imaging (left) and quantitation (right) of PLA signals for NONO/RNAPII or NONO/SPT5. Each dot represents one acquisition. n, number of cells; a.u., arbitrary units. (**B**) NONO CUT&RUN-seq at the TSS of the top 1000 expressed genes. Red, promoter region. (**C**) Browser tracks of NONO and histone H3 Lys4 tri-methylation (H3K4me3) CUT&RUN-seq. Red, promoter region. (**D**) 4sU-seq read counts for the gene body of 863 highly expressed genes. *P-value < 0.05; **P-value < 0.001; two-tailed *t*-test. Error bar, mean ± SD. Representative images are shown. n = number of biological replicates.

DSB signalling inhibits RNAPII activity, in particular close to TSSs, and NONO stimulates pre-mRNA synthesis in unperturbed cells (44,45). Thus, we speculated that the etoposide-induced nucleolar re-localization of NONO coincides with reduced RNAPII activity on broken chromatin. To test this, we performed PLAs for NONO and RNAPII or the elongation factor SPT5 and observed prominent PLA signals for both reactions in unperturbed cells, which were sensitive to etoposide treatment (Figure 3A). Thus, we investigated if DNA damage alters NONO chromatin occupancy at protein-coding genes and performed CUT&RUN-seq with the NONO antibody. We observed prominent binding of NONO in a region from the TSS up to 1 kb downstream of the TSS, but not in the gene body or the transcription end site of highly expressed genes, which was markedly reduced upon etoposide treatment and sensitive to NONO depletion (Figure 3B, C; Supplementary Figure S8A, B). We validated CUT&RUN-seq data by NONO ChIP assays and detected NONO occupancy downstream of the TSSs of *ACTB* and *CCNB1*, which was sensitive to etoposide treatment (Supplementary Figure S8C). Next, we applied 4sU-seq to measure nascent RNA synthesis. We found that etoposide treatment reduced the bulk of pre-mRNA synthesis on average by 25% within the gene body of highly expressed genes (Figure 3D; Supplementary Figure S8D). The depletion of NONO *per se* reduced 4sU-seq reads to a similar extent, but the reads were not further reduced by combining NONO depletion with etoposide incubation. Thus, etoposide treatment depletes NONO from the promoter-proximal region of some highly expressed genes to attenuate RNAPII activity.

### NONO mediates the accumulation of pre-mRNA transcripts in the nucleolus

**Figure 4.**
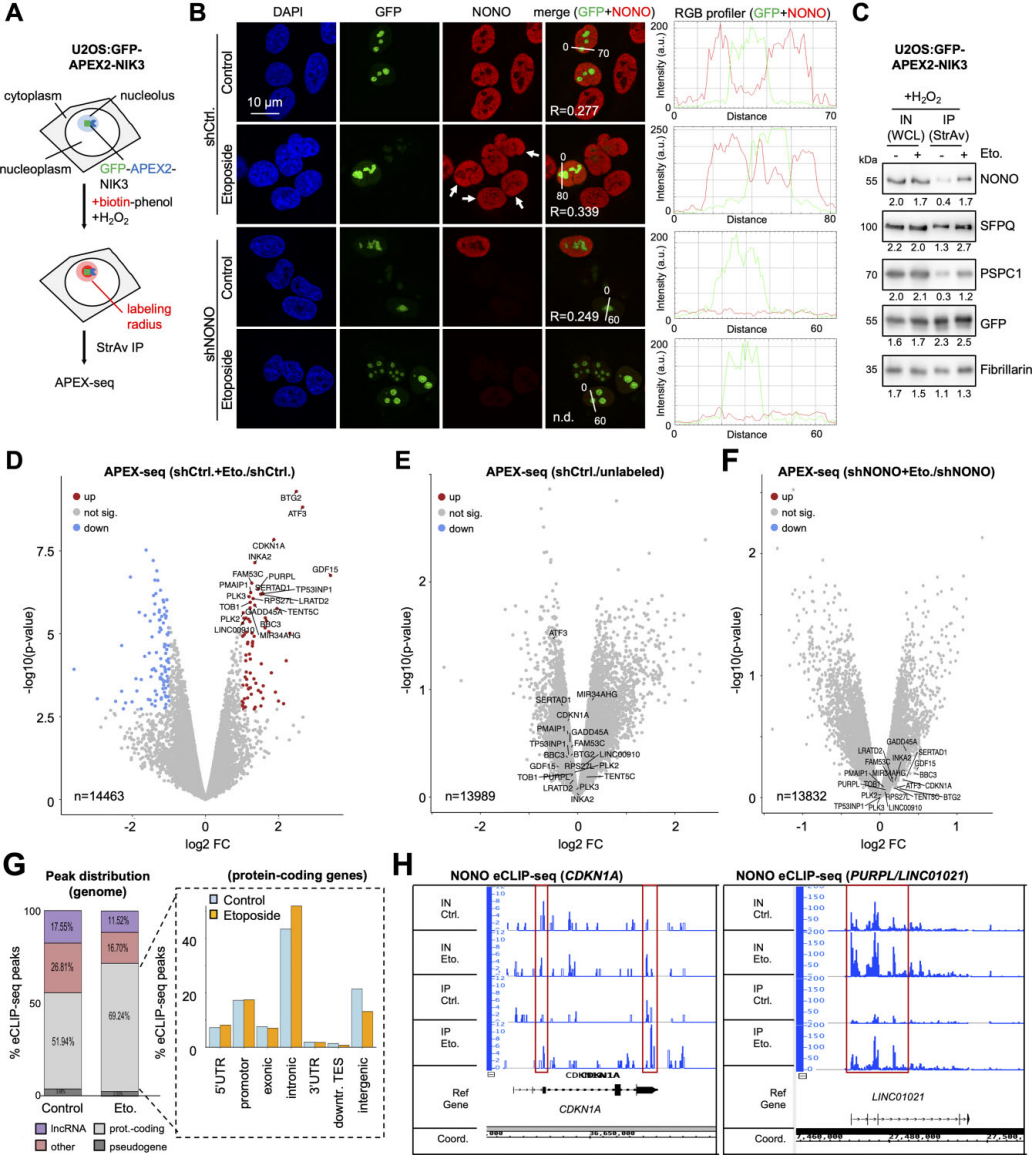
NONO mediates the nucleolar accumulation of transcripts in U2OS cells. (**A**) Schematic displaying APEX-seq in U2OS:GFP-APEX2-NIK3 cells. StrAv, streptavidin; H_2_O_2_, hydrogen peroxide. (**B**) Imaging (left) and RGB profiler line scans (right) of GFP and NONO in U2OS:GFP-APEX2-NIK3 cells. R = Pearson correlation; n.d., not detected; arrowhead, pan-nuclear NONO. Representative images are shown. (**C**) Immunoblots detecting NONO, SFPQ, PSPC1, GFP–APEX2-NIK3 and fibrillarin upon incubation with biotin–phenol, H_2_O_2_ from whole-cell lysates (WCLs) or upon immunoselection with streptavidin-coated beads. (**D–F**) Volcano plots displaying the relative abundance of transcripts as ratios of reads. Red, over-represented; blue, under-represented; n = number of transcripts. (**G**) NONO eCLIP-seq peak distribution genome wide (left) and at the gene body (right). (**H**) Browser tracks for NONO eCLIP-seq reads at the *CDKN1A* (left) and *PURPL* (right) locus. Red, increased binding.

NONO preferentially binds intron-containing transcripts in unperturbed cells (46,47). We reasoned that DDR shifts NONO from protein-coding chromatin to the nucleolus to detain nascent transcripts from broken chromatin. To explore DNA damage-induced NONO-dependent changes in the nucleolar transcriptome, we created U2OS cells that stably express a GFP-tagged ascorbate peroxidase 2 fused with three nucleolar targeting sequences from the NF-κB-inducing kinase (U2OS:GFP-APEX2-NIK3), which can be used to map nucleolar transcripts *in vivo* by proximity labelling and subsequent sequencing of immunoselected biotinylated RNA (APEX-seq) (48) (Figure 4A). We confirmed GFP–APEX2-NIK3-mediated biotinylation of nucleic acids by dot blotting (Supplementary Figure S9A) and validated the selective biotinylation of RNA on agarose gels by immunoselection and RNase A digestion (Supplementary Figure S9B). We further confirmed nucleolar localization and activity of the GFP–APEX2-NIK3 reporter irrespective of etoposide treatment by co-staining with NCL and a fluorescently labelled neutravidin probe (Supplementary Figure S9C, D). Importantly, the expression of GFP–APEX2-NIK3 did not interfere with NONO nucleolar re-localization, nor did the depletion of NONO interfere with GFP–APEX2-NIK3 localization (Figure 4B; Supplementary Figure S9E). Reassuringly, we found that proximity-mediated biotinylation by nucleolar GFP–APEX2-NIK3 in the presence of etoposide increased the amount of DBHS proteins that co-immunoprecipitate with streptavidin beads 2- to 4-fold, but not the amount of GFP–APEX2-NIK3 or fibrillarin (Figure 4C). This prompted us to perform APEX-seq in U2OS:GFP-APEX2-NIK3 cells. By assessing fold changes for a total of 14 463 intron-containing, biotin-labelled transcripts, we found 75 candidates with significantly higher biotinylation upon etoposide treatment (Figure 4D). To exclude that the changes in the levels of biotinylated transcripts reflect lentiviral stress or perturbations upon biotin–phenol/H_2_O_2_ treatment, we compared the ratios of labelled transcripts from lentiviral-transduced cells with rRNA-depleted transcripts immunoselected from unlabelled and unperturbed controls. We found no significant changes in the levels of biotinylated transcripts (Figure 4E). To assess if the differential biotinylation of transcripts depends on NONO, we repeated APEX-seq upon NONO depletion. Strikingly, NONO depletion abolished the differential biotinylation, but not the synthesis of most candidates (Figure 4F; Supplementary Figure S9F). To assess if NONO binds candidates differentially upon DNA damage, we employed the NONO antibody for eCLIP-seq. We found that etoposide increased the total number of NONO eCLIP-seq peaks from 2649 to 3991 and particularly enhanced NONO binding to intron-containing transcripts, including the previously identified transcript DAZAP1 (47), and some of the identified APEX-seq candidates (CDKN1A, PURPL) (Figure 4G, H; Supplementary Figure S9G). This suggests that NONO mediates nucleolar detention of a subset of pre-mRNA transcripts upon DNA damage.

### NONO binds CDKN1A transcripts to suppress R-loops at the *CDKN1A* locus

**Figure 5.**
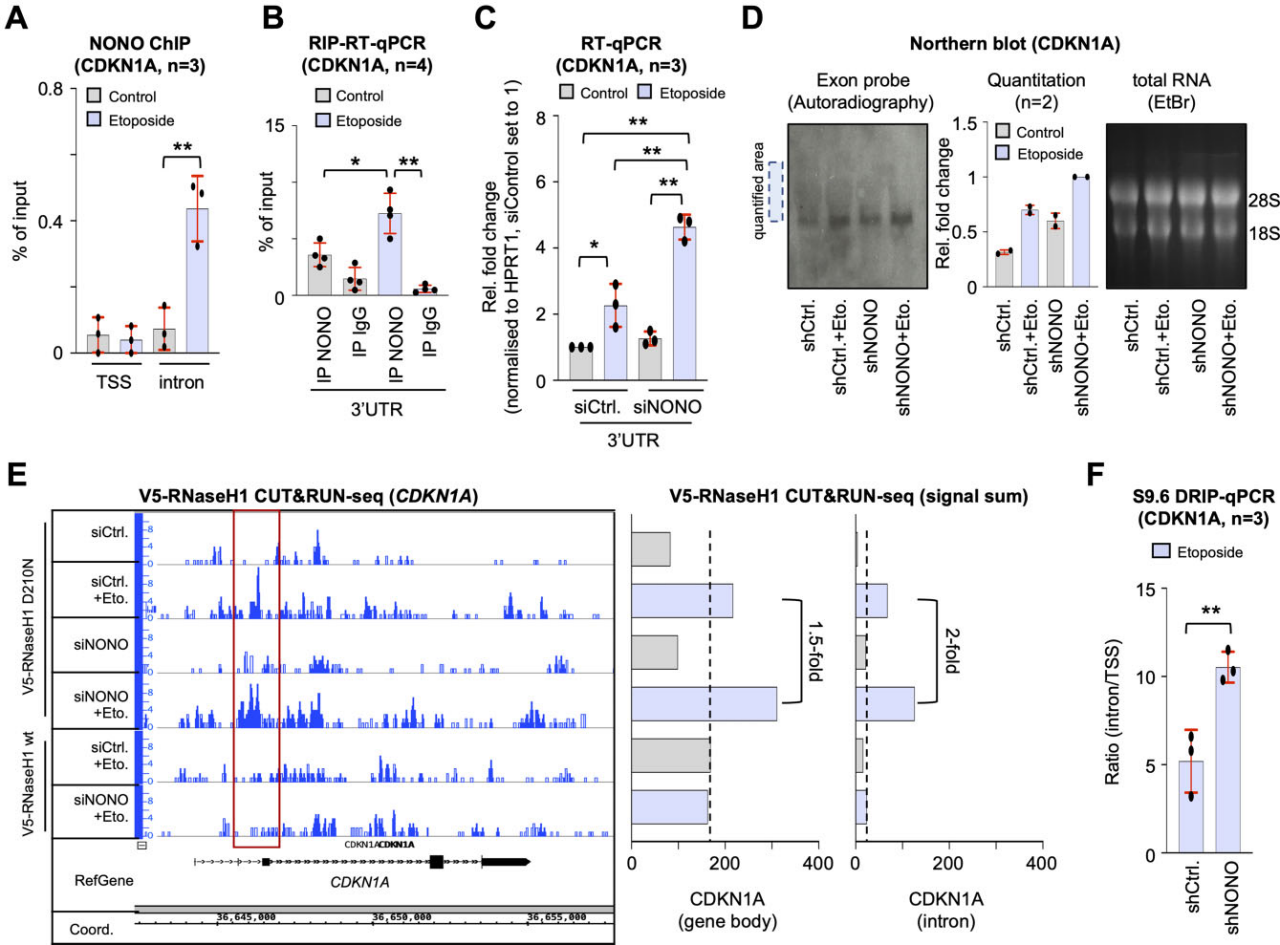
NONO regulates transcript levels at the *CDKN1A* locus in U2OS cells. (**A**) NONO ChIP using site-specific primers. (**B**) RIP–RT–qPCR using region-selective primers. IgG, immunoglobulin, control IP. (**C**) RT–qPCR using site-specific primers ± NONO depletion/etoposide. (**D**) Autoradiograph (left) and quantitation (middle) displaying signals upon northern blot hybridization using region-specific, radiolabelled CDKN1A exon probe. EtBr, ethidium bromide loading control (right); dotted box, area of quantification. (**E**) Browser tracks (left) and quantitation (right) depicting V5-RNaseH1 CUT&RUN-seq reads for *CDKN1A*± NONO depletion/etoposide. Red box, region of increase; dashed line, background. (**F**) S9.6 DRIP using site-specific primers. Signals are shown as ratios of the percentage of inputs from the CDKN1A intronic site over the TSS site. *P-value < 0.05; **P-value < 0.001; two-tailed *t*-test. Error bar, mean ± SD. n = number of biological replicates.

Our APEX-seq and eCLIP-seq data both point toward etoposide-responsive binding of NONO to a subset of pre-mRNA transcripts for subsequent nucleolar detention. We focused on the *CDKN1A* gene, one of the top hits in the APEX-seq experiment, and asked if we could derive a NONO-dependent pathway that controls genome stability at the *CDKN1A* locus. First, we assessed NONO occupancy at the *CDKN1A* locus. We used ChIP with primers that target the TSS of *CDKN1A* or the intronic region that displayed elevated NONO binding in our eCLIP-seq data (Supplementary Figure S10A). Whilst we detected no significant antibody reactivity at the TSS of *CDKN1A*, etoposide treatment increased NONO occupancy at the intronic region (Figure 5A). When reassessing our NONO CUT&RUN-seq data, we could indeed confirm the appearance of an etoposide-responsive NONO peak, which was sensitive to NONO depletion, in this intronic region (Supplementary Figure S10B). Next, we immunoselected NONO from cells cultured in the presence or absence of etoposide and quantified the amount of CDKN1A mRNA bound to NONO (Figure 5B; Supplementary Figure S10C). We found significant binding of such transcripts in the presence of etoposide only. Next, we depleted NONO by RNA interference (RNAi) and assessed the level of CDKN1A mRNA transcripts by RT–qPCR and northern blot hybridization in the absence or presence of etoposide (Figure 5C, D; Supplementary Figure S10D). As expected, the combination of NONO depletion and etoposide treatment significantly elevated the level of CDKN1A mRNA, whilst etoposide treatment or NONO depletion alone displayed little change in transcript levels compared with unperturbed cells. NONO binding to chromatin-associated transcripts is strongly correlated with the formation of R-loops (49). Thus, we tested if defects in nucleolar detention observed in NONO-deficient cells may be linked to aberrant R-loop levels at the *CDKN1A* locus. Therefore, we transiently expressed the R-loop-stabilizing V5-tagged RNase H1 D210N mutant, or wild-type control in the absence or presence of etoposide (Supplementary Figure S10E). To assess R-loop levels, we employed the V5 antibody in CUT&RUN-seq. We found that the depletion of NONO prior to etoposide treatment increased the levels of R-loops at the CDKN1A locus, in particular within the exact same intronic region of *CDKN1A* (Figure 5E). We confirmed this phenotype by S9.6 DRIP at the *CDKN1A* locus (Figure 5F). This suggests that NONO binds etoposide-responsive CDKN1A transcripts to mediate nucleolar detention and mitigate R-loop levels upon DNA damage at the *CDKN1A* locus.

### NONO inactivation impairs DSB signalling

**Figure 6.**
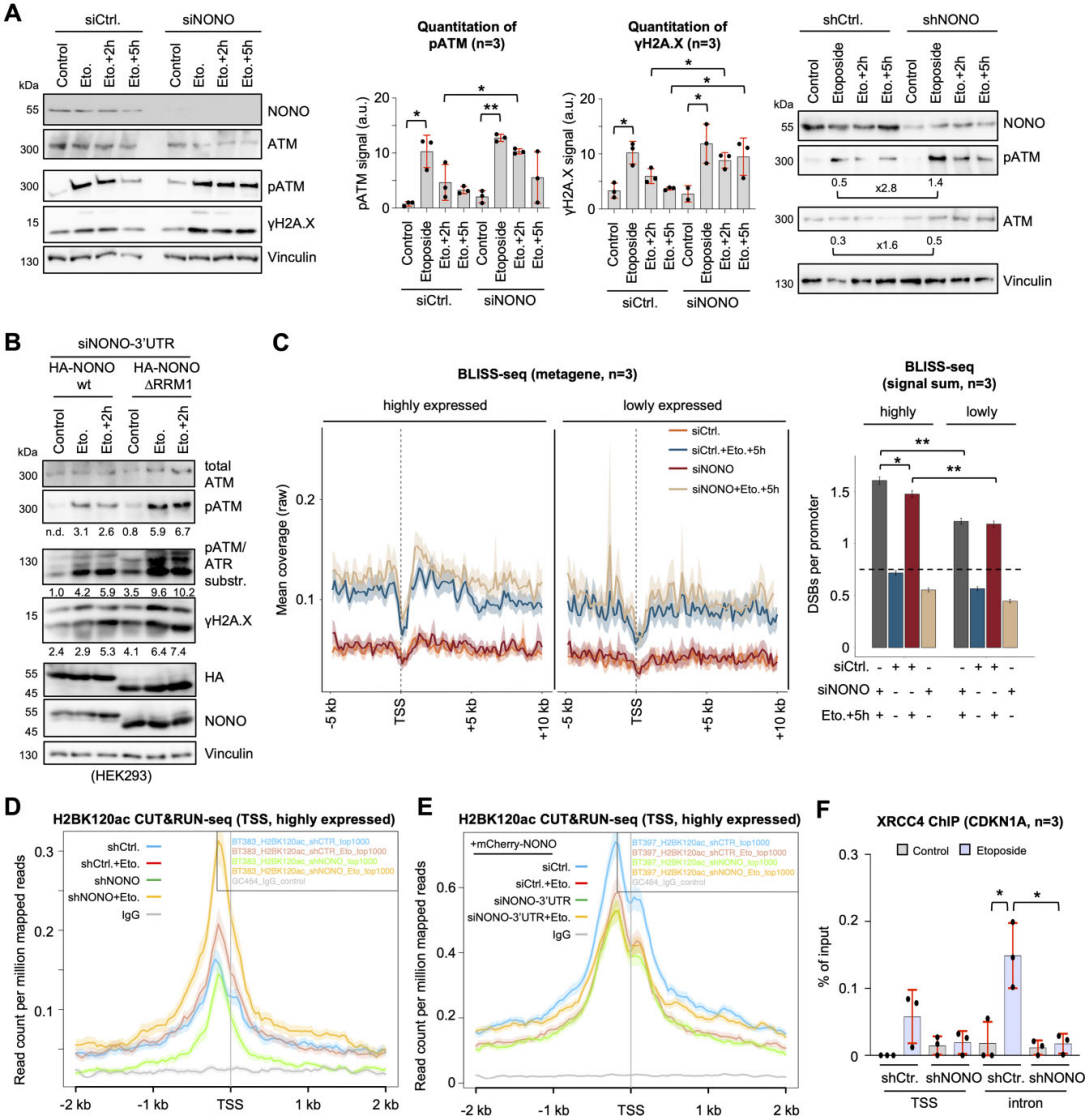
Impairment of NONO interferes with DSB signalling in U2OS cells. (**A**) Immunoblots (left) and quantitation (middle) of NONO, total ATM, phospho-(p)ATM and γH2A.X ± NONO depletion/etoposide via siRNA (left, middle) or shRNA (right). Vinculin, loading control; a.u., arbitrary units. (**B**) Immunoblots detecting total ATM, pATM, pATM/ATR substrates, γH2A.X, HA-NONO and endogenous NONO upon depletion of endogenous NONO and re-expression of HA-NONO variants. (**C**) BLISS-seq metagene profiles (left) and signal sum (right) detecting DSBs at the TSS of genes which are expressed at high and low levels. Dashed line, background. (**D** and **E**) CUT&RUN-seq metagenes displaying histone H2B Lys120 acetylation (H2BK120ac) chromatin occupancy at TSSs of the top 1000 highly expressed genes ± NONO depletion/etoposide (D) and upon re-expression of mCherry–NONO (E). (**F**) XRCC4 ChIP using site-specific primers ± NONO depletion/etoposide. *P-value < 0.05; **P-value < 0.001; two-tailed *t*-test. Error bar, mean ± SD. n = number of biological replicates.

NONO depletion elevates R-loop levels at telomeres and promotes genome instability (50). To test the impact of NONO depletion on DSB signalling, we performed etoposide incubation kinetic experiments and detected defects in clearing Ser1981-phosphorylated (p)ATM and γH2A.X upon NONO depletion using either siRNA or shRNA (Figure 6A). As expected, the siRNA-mediated depletion of NONO had no significant impact on cell cycle progression (Supplementary Figure S11A) and complementation with mCherry–NONO rescued γH2A.X levels partially (Supplementary Figure S11B). To investigate if the DDR function of NONO may be linked to its nucleolar re-localization, we assessed the impact of RRM1 depletion on DSB signalling. We found that overexpression of ΔRRM1, but not FL RRM1, increased phosphorylation of DDR markers (Figure 6B). Next, we asked if NONO depletion elevates the amount of DSBs and used BLISS-seq to quantify DSBs (Figure 6C). Indeed, NONO depletion prior to etoposide treatment increased the amount of DSBs compared with non-depleted, etoposide-treated cells at TSSs of highly expressed genes. For validation, we performed γH2A.X ChIP at the AsiSI site DS1 (Supplementary Figure S11C). Again, NONO depletion prior to 4-OHT incubation increased γH2A.X levels ∼2-fold. Interestingly, histone H2B acetylation at Lys120 residues (H2BK120ac) functions as a chromatin switch during DSB repair at AsiSI sites (42). Thus, we applied CUT&RUN-seq to quantify the levels of H2BK120ac at TSSs of highly expressed genes such as *CHD4* (Figure 6D; Supplementary Figure S11D). The depletion of NONO or etoposide treatment alone modestly altered H2BK120ac levels at TSSs. Combining NONO depletion with etoposide treatment, however, strongly increased the H2BK120ac mark not only at the TSSs of highly expressed genes, but also at the aforementioned intronic *CDKN1A* locus (Supplementary Figure S11E). We also rescued elevated H2BK120ac levels by re-expression of mCherry–NONO (Figure 6E). To test if NONO-dependent suppression of R-loops at the *CDKN1A* locus could be coupled to the recruitment of DSB repair factors, we assessed the chromatin occupancy of the NHEJ factor XRCC4. NONO promotes DSB repair via NHEJ and has previously been described as a stimulator of XRCC4 (51,52). Our ChIP data suggest that etoposide treatment elevates the levels of XRCC4 at the intronic CDKN1A region, whilst NONO depletion prior to etoposide incubation prevents the recruitment of XRCC4 to that site (Figure 6F). We conclude that NONO inactivation impairs DSB repair at a subset of highly expressed genes and at the *CDKN1A* locus.

## Discussion

**Figure 7.**
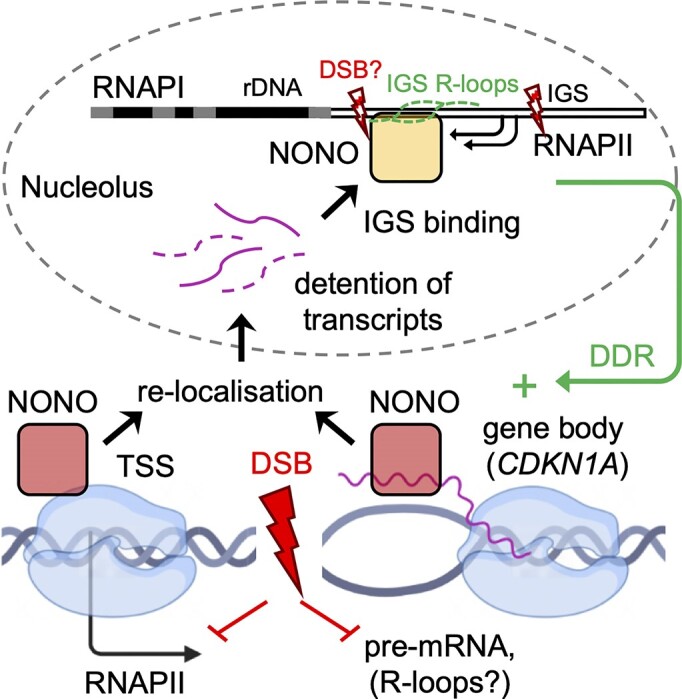
Model illustrating our findings. See main text for details.

We describe NONO as attenuator of pre-mRNA synthesis and a nucleolar detainer of nascent transcripts to promote DSB repair (Figure 7). Our data suggest a two-pronged attack of NONO on nascent pre-mRNA synthesis upon DNA damage. Firstly, NONO displays comprised occupancy on the TSS of highly expressed genes to reduce RNAPII activity. Secondly, NONO shows increased binding to a subset of pre-mRNA transcripts (e.g. CDKN1A) to mediate nucleolar detention of the latter. Both modes of NONO action impair the formation of excessive R-loops and facilitate efficient NHEJ. It is important to stress that these two modes of NONO action do not necessarily represent a linear pathway that works on the same gene, but may rather co-exist and support each other upon DNA damage by targeting R-loop-prone loci both *in cis* and *in trans*.

Many RBPs display stress-induced nucleolar re-localization (39,40). We provide evidence for asincRNA-induced IGS R-loops as an anchor for nucleolar NONO. How is asincRNA synthesis regulated? We show that the increase of RNAPII CTD S2P occupancy at asincRNA-encoding IGS loci correlates with elevated levels of H3K4me3 modifications. Several writers and erasers for H3K4me3 marks have been described in human cells. Intriguingly, the lysine methyltransferase 2B (KMT2B/MLL2) is a direct target of ATM and its mutation results in elevated levels of transcription stress, DNA damage and genome instability (53,54). It is tempting to speculate that the accumulation of H3K4me3 marks within the IGS involves DNA damage-responsive activation of KMT2B/MLL2 activity, probably in concert with additional yet to be described factors. A subset of RNAPII elongation factors may also be enriched in the nucleolus, as shown for Spt4 in yeast, for instance (55).

Interestingly, recent data using targeted DSB induction within rDNA suggest that the formation of nucleolar caps and subsequent repair of broken rDNA with such caps requires RNAPII activity (57). Likewise, IGS loci may become accessible for RNAPII upon etoposide treatment, which does not trigger the formation of nucleolar caps, by looping of nucleolar DNA to the nucleoplasm. Alternatively, etoposide may also stimulate RNAPII activity by impacting on RNAPI. DSB signalling indeed attenuates RNAPI transcription via ATM when DSBs occur both in the nucleolus and in the nucleoplasm (57–60). Nucleoplasmic DSBs activate ATM and can cause a global and transient inhibition of RNAPI activity *in trans*. The induction of nucleolar DSBs also correlates with a transient, locally restricted ATM- and ATR-dependent inhibition of RNAPI *in cis*, which subsequently and upon persistent rDNA damage and more prominent RNAPI inhibition leads to nucleolar segregation, and translocation of nucleolar proteins to the nucleoplasm or accumulation in nucleolar caps (58). We assessed the localization of NPM1 and TCOF1 upon etoposide treatment and found that short-term (2 h) treatment with 20 μM etoposide induced neither prominent NPM1 translocation nor TCOF1-positive nucleolar caps. Both phenotypes could, however, be prominently observed upon prolonged etoposide treatment with a higher concentration (4 h, 100 μM). We further detected TOPBP1-positive nucleolar caps after targeted persistent induction of DSBs with I-PpoI and, to a lesser degree, AsiSI, around round-shaped (i.e. disintegrated, rDNA damaged) nucleoli. Short-term treatment with etoposide, in contrast, induced TOPBP1 foci throughout the nucleoplasm without particular enrichment around nucleoli, which also remained mostly amorphic in shape (i.e. not disintegrated, not severely damaged). Moreover, NONO nucleolar relocalization into round-shaped nucleoli could not be observed upon either I-PpoI or AsiSI cleavage, or upon targeted induction of nucleolar DSBs via CRISPR/Cas9. Thus, our etoposide-induced imaging phenotype differs from the phenotype induced by the endonucleases I-PpoI and AsiSI, but could be confirmed upon DSBs in 5S loci. The behaviour of the nucleolus upon etoposide treatment is somewhat surprising, as many topoisomerase inhibitors are known to potently induce DNA damage, inhibit RNAPI and trigger nucleolar disintegration. We could previously show that etoposide inhibits RNAPI but only after a prolonged treatment time and at concentrations > 50 μM (61,62). Recent data underscore that etoposide may be a somewhat exceptional topoisomerase inhibitor (63). The authors tested the impact of various topoisomerase inhibitors on nucleolar integrity, RNAPI activity and rDNA damage, and could show that etoposide, doxorubicin, aclarubicin as well as DNA topoisomerase I inhibitors (camptothecin and topotecan) are all inducing markers of DNA damage and are all destabilizing topoisomerase II (TOP2). However, only etoposide (50 μM) did not inhibit RNAPI and did not trigger a nucleolar disintegration phenotype. The authors eventually observed nucleolar segregation, but still no inhibition of RNAPI, after combining etoposide treatment with depletion of TDP-2, an enzyme that removes trapped TOP2 from the DNA end (64), probably due to induction of more persistent DNA damage. Etoposide acts as a pure TOP2 poison that stabilizes the TOP2–DNA covalent complexes and protects the re-ligation of DNA. Thus, the formation of DSBs is the final consequence of etoposide exposure. Other topoisomerase inhibitors such as doxorubicin not only stabilize the TOP2–DNA complex, but also inhibit the decatenation, intercalate with DNA and, by this, alter DNA torsion and even induce histone eviction and elevated oxidative stress. In a nutshell, the DNA damage introduced by doxorubicin is more complex than etoposide-induced DSBs.

Based on this, we postulate that DSBs introduced by etoposide are different from those by AsiSI or I-PpoI cleavage, as they do not cause sufficient targeted, locus-specific lesions within (r)DNA to interfere with nucleolar RNA synthesis and integrity. We hypothesize that the non-selective induction of DSBs by low-dose/short-term etoposide treatment (as used throughout the study) includes mostly nucleoplasmic and some modest amount of nucleolar DSBs that may be regarded as easy-to-repair, non-persistent lesions (i.e. TOP2 adducts trapped by etoposide), which are rapidly cleared by TDP-2. Thus, etoposide treatment may result in a transient and locally restricted inhibition of RNAPI without nucleolar disintegration. Of note, a similar phenotype has also been observed upon targeted ionizing radiation (60,65). This, and the relatively modest reduction of pre-rRNA levels observed upon etoposide treatment, suggests that etoposide does not severely impair, but rather attenuates RNAPI, which favours the synthesis of asincRNA and the re-localization of NONO to non-disintegrated nucleoli in response to predominantly nucleoplasmic DSBs that trigger a sufficiently strong DSB signalling to exceed a yet to be defined threshold required for the activation of the nucleolar detention pathway.

We observed a rapid decrease in NONO chromatin occupancy downstream of some protein-coding gene promoters within 2 h of etoposide treatment, whilst the formation of IGS R-loops was stable upon chase, and NONO accumulation at nucleolar IGS loci could prominently be detected upon chase only. Thus, the reduction of NONO at promoter regions probably precedes its re-localization to nucleoli and impairs pre-mRNA synthesis as a consequence thereof. Early studies identified NONO as a transducer of cAMP signalling that interacts with the CBP/p300 co-activator complex, co-purifies with the mediator complex and associates with the RNAPII CTD (66–68). NONO is indeed enriched in condensates to enhance the expression of pre-mRNA transcripts *in vitro* and *in vivo* (47,69). Other DBHS proteins also stabilize nascent pre-mRNA and favour the placement of RNAPII-activating CTD phospho-marks at promoters (70). This suggests that DBHS proteins foster RNAPII activity and that NONO nucleolar re-localization diminishes RNAPII-stimulating conditions.

We find that NONO nucleolar re-localization attenuates pre-mRNA synthesis to mitigate aberrant transcripts via nucleolar shielding, for instance at the *CDKN1A* locus. This pathway could promote R-loop-dependent DSB repair pathway choice. R-loops accumulate at actively transcribed DSBs (71). R-loops foster the recruitment of critical homologous recombination factors to DSBs (72,73). R-loops also promote DNA end resection via DNA endonuclease CtIP, a critical step in DSB repair pathway choice (74). As exemplified for the *CDKN1A* locus in this study, the NONO-mediated nucleolar detention of transcripts may suppress R-loops and, at least in part, explain promotion of NHEJ by NONO (20,51). Overall, we provide evidence that the DDR engages NONO to shield aberrant transcripts from DSBs.

Our conclusions are based on experiments performed in tissue culture, using predominantly U2OS cells as a model system and etoposide as DSB-inducing agent to study the DDR. Albeit widely used in the field, it remains to be determined to what extent our proposed pathway may be conserved in a multicellular system and utilized for the maintenance of genomes upon physiological endogenous DSB-inducing DNA damage, such as the formation of transcription–replication conflicts. Several studies suggest that NONO preferentially binds to intron-containing transcripts (75,76), and our APEX-seq and eCLIP-seq data point toward a NONO-dependent nucleolar accumulation of nascent transcripts. Our model predicts elevated levels of nucleolar pre-mRNA transcripts in response to DNA damage. However, the panel of proximity labelled candidates is rather limited, with no clear enrichment of a distinct class of transcripts and no obvious correlation with expression levels, except many of the top hits being p53 target genes. This may point toward several interesting features of the pathway: after being released from the TSSs, NONO may bind nascent pre-mRNA for nucleolar detention both *in cis* and *in trans*. Nucleolar detention of such transcripts could, for instance, require NONO post-translational modifications or the recognition of distinct RNA-binding motifs. The nucleolus probably promotes the turnover of detained transcripts, as recently demonstrated upon viral infection (77), but may also regulate the stability of transcripts upon nucleolar transit, for instance by mediating the placement of epitranscriptomic marks that foster translation upon a transient and reversible re-localization of NONO. This may apply in particular for transcripts involved in the metabolism of p53, as the nucleolus is a well-established regulator of p53 response. It will be important to address these possibilities in future studies.

## Supplementary Material

gkae022_Supplemental_File

## Data Availability

Sequencing data are available at the Gene Expression Omnibus under the accession numbers GEO:GSE236900 and GEO:GSE233594, and are viewable on the integrated genome browser (IGB) or other suitable genome browsers. Further information and requests for resources and reagents should be directed to and will be fulfilled by the corresponding author.

## Notes

Present address: Apoorva Baluapuri, Department of Biological Chemistry and Molecular Pharmacology, Blavatnik Institute, 45 Shattuck St., Harvard Medical School, Boston, MA, USA.
