# Supplementary material for: Nucleolar detention of NONO shields DNA double-strand breaks from aberrant transcripts
Source: Nucleic Acids Res. 2024 Jan 15;52(6):3050–68. doi: 10.1093/nar/gkae022 (PMC11014278; doi:10.1093/nar/gkae022)
Supplement: gkae022_Supplemental_File [file gkae022_supplemental_file.pdf]

SUPPLEMENTARY DATA for

Nucleolar detention of NONO shields DNA double-strand breaks from aberrant transcripts

AUTHORS

Barbara Trifault[1,2], Victoria Mamontova[1,2], Giacomo Cossa[2], Sabina Ganskih[3], Yuanjie Wei[3], Julia Hofstetter[4], Pranjali Bhandare[4], Apoorva Baluapuri[4], Blanca Nieto[5], Daniel Solvie[2], Carsten P. Ade[2], Peter Gallant[2], Elmar Wolf[4], Dorthe H. Larsen[5], Mathias Munschauer[3], and Kaspar Burger[1,2,6]*

[1]Mildred Scheel Early Career Center for Cancer Research (Mildred-Scheel-Nachwuchszentrum, MSNZ) Würzburg, University Hospital Würzburg, Josef-Schneider-Str. 2, 97080 Würzburg, Germany.

[2]Department of Biochemistry and Molecular Biology, Biocenter of the University of Würzburg, Am Hubland, 97074 Würzburg, Germany.

[3]Helmholtz Institute for RNA-based Infection Research, Helmholtz-Center for Infection Research, Josef-Schneider-Str. 2, 97080 Würzburg, Germany.

[4]Cancer Systems Biology Group, Theodor Boveri Institute, Biocenter, University of Würzburg, Am Hubland, 97074 Würzburg, Germany.

Present Address: Apoorva Baluapuri, Department of Biological Chemistry and Molecular Pharmacology, Blavatnik Institute, 45 Shattuck St., Harvard Medical School, Boston, MA, USA.

[5]Nucleolar Stress and Disease Group, Danish Cancer Institute, Strandboulevarden 49, Copenhagen, Denmark.

[6]Lead contact.

*To whom correspondence should be addressed: Tel: ++49-931-31-48975; Email: kaspar.burger@uni-wuerzburg.de

Supplementary data contains 11 supplementary figures and 8 supplementary tables.

Supplementary Figure 1

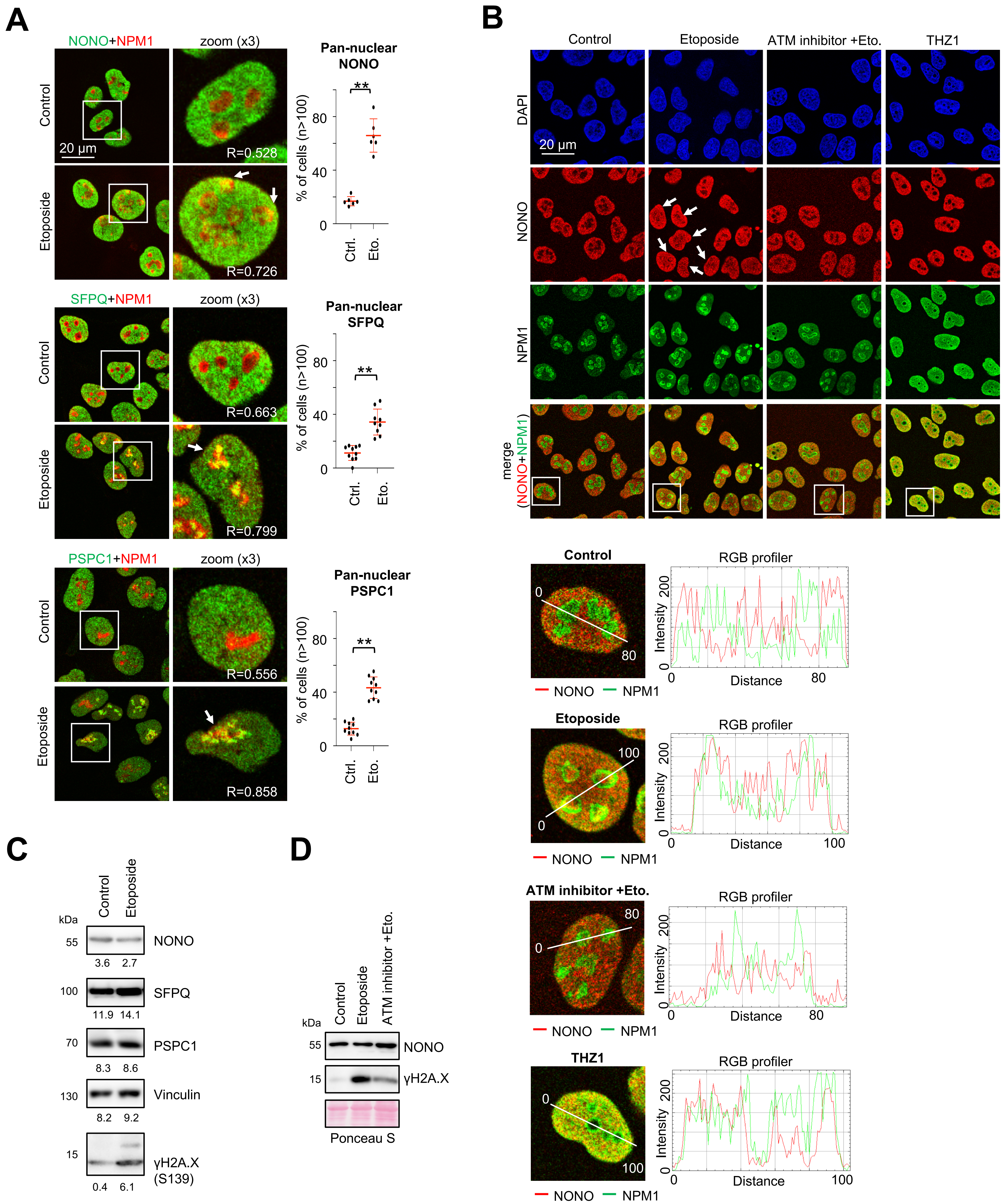

Supplementary Figure 1. Validation of NONO nucleolar re-localisation upon DNA damage in U2OS cells. (**A**) Imaging (left) and quantitation (right) of NONO, splicing factor proline and glutamine rich (SFPQ), and paraspeckle component 1 (PSPC1) with nucleophosmin (NPM1) in the absence or presence (±) of etoposide. White box, zoom; arrowhead, colocalisation; R=Pearson correlation; n, number of cells. Each dot represents % of cells with pan-nuclear signals as average from one acquisition. *, p-value <0.05; **, p-value <0.001; two-tailed t-test. Error bar, mean ±SD. (**B**) Imaging (top) and line scan quantitation of colocalisation (bottom) of NONO and NPM1 ±etoposide or pretreatment with ATM inhibitor or incubation with THZ1. Arrowhead, pan-nuclear localisation. (**C, D**) Immunoblots detecting NONO, SFPQ, PSPC1 and ser-139 phosphorylated histone H2A.X variant (γH2A.X). Vinculin and Ponceau S, loading controls. Representative images are shown.

Supplementary Figure 2

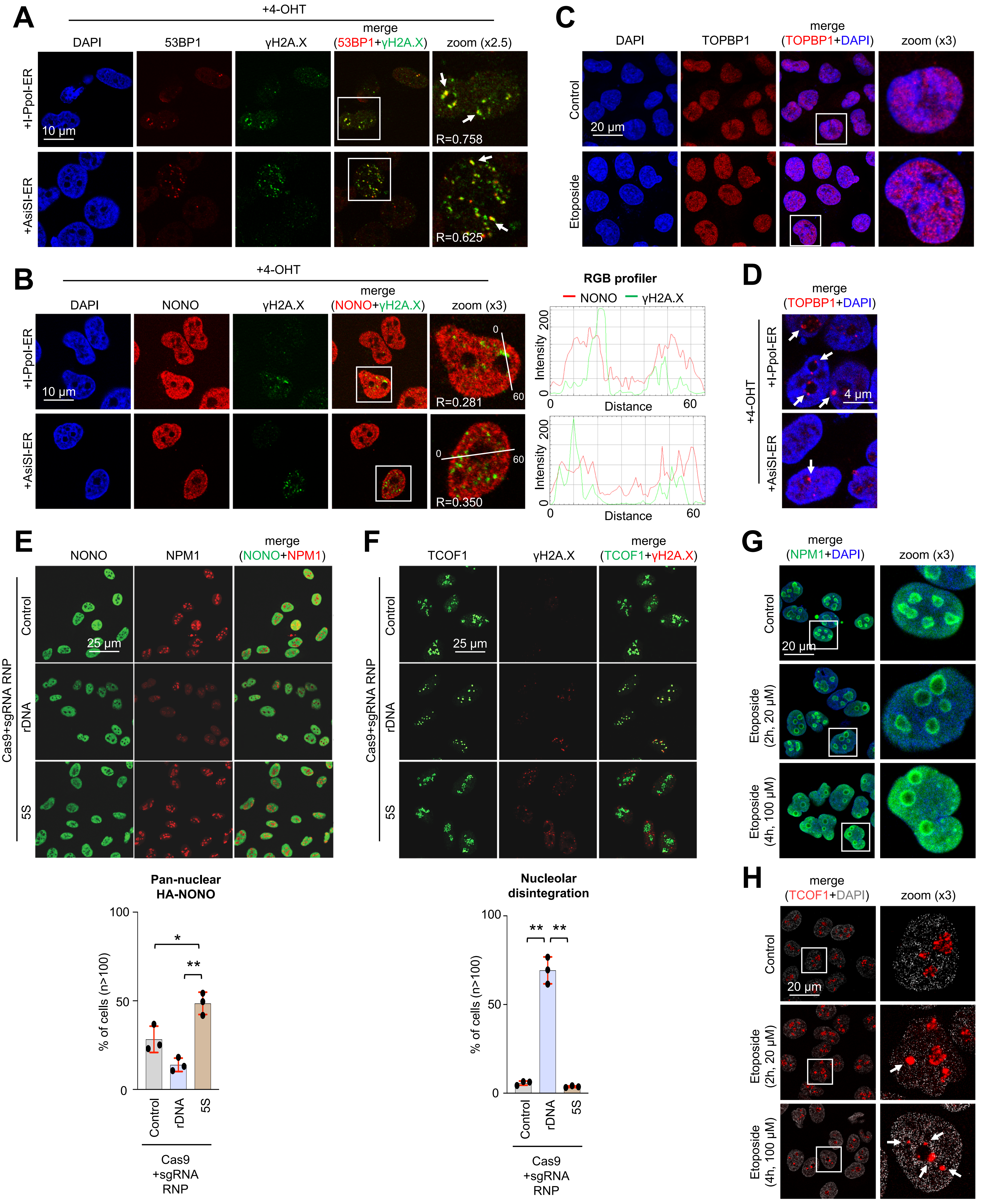

Supplementary Figure 2. Assessment of NONO localisation upon induction of locus-specific DSBs and persistent etoposide treatment in U2OS cells. (**A, B**) Imaging of 53BP1 and γH2A.X (A) or NONO and γH2A.X (B, left) and quantitation of colocalisation (B, right) upon transfection of I-PpoI- or AsiSI-encoding plasmids in U2OS cells in the presence of 4-hydroxytamoxifen (4-OHT). White box, zoom; arrowhead, colocalisation; R=Pearson correlation. (**C, D**) Imaging of TOPBP1 ±etoposide (C) or upon transfection of I-PpoI- or AsiSI-encoding plasmids and incubation with 4-OHT (D). White box, zoom; arrowhead, nucleolar caps. (**E, F**) Imaging (top) and quantitation (bottom) of NONO and NPM1 (E) or TCOF1 and γH2A.X (F) upon transfection of Cas9-synthetic guide (sg)RNA ribonucleoprotein complexes targeting nucleolar (rDNA) or nucleoplasmic (5S) sequences. (**G, H**) Imaging of NPM1 (G) or TCOF1 (H) ±etoposide. White box, zoom; arrowhead, nucleolar caps. *, p-value <0.05; **, p-value <0.001; one-way Anova with Tukey´s post hoc test. Error bar, mean ±SD. Representative images are shown.

Supplementary Figure 3

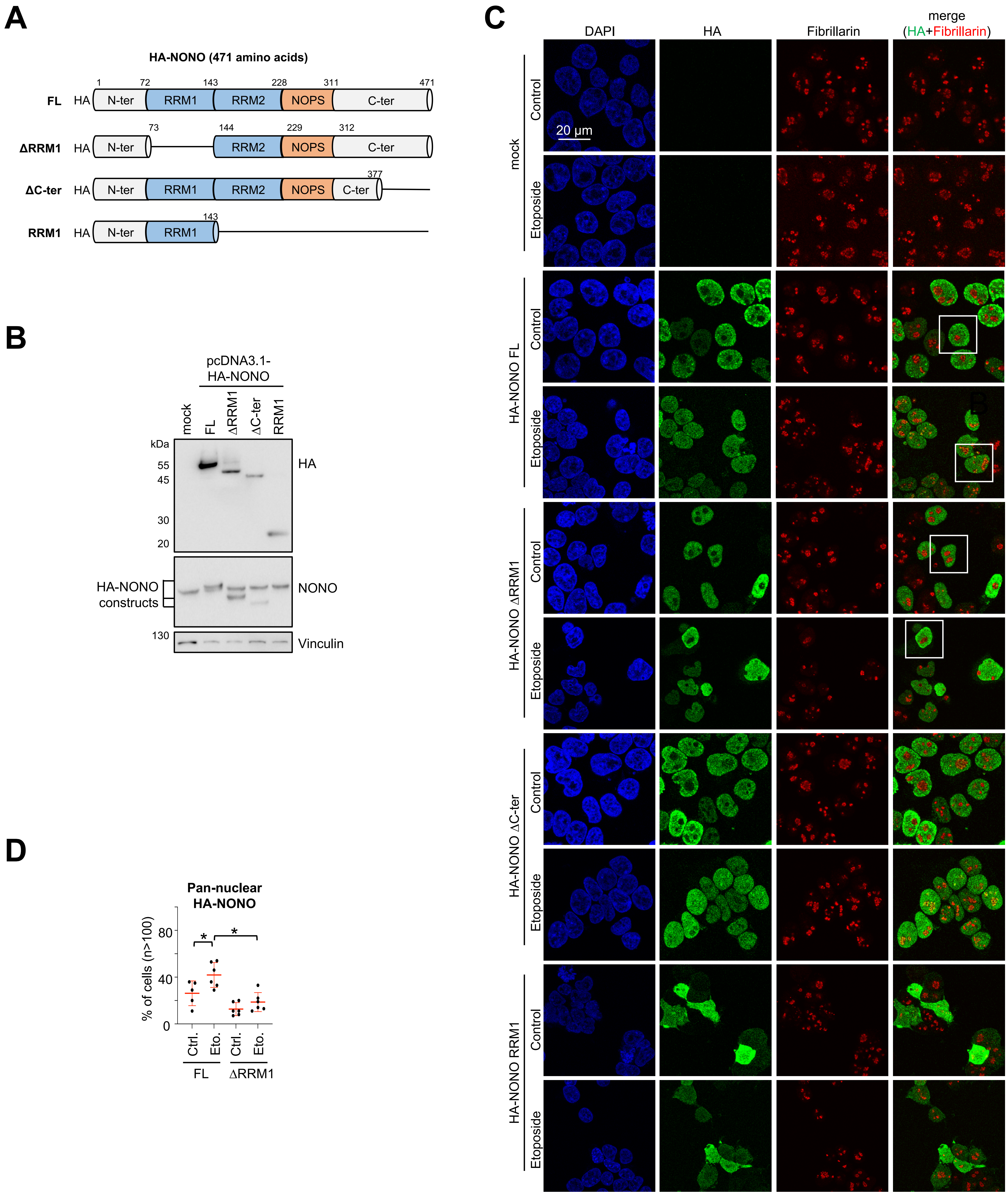

Supplementary Figure 3. Assessment of HA-NONO constructs upon etoposide treatment in U2OS cells. (**A**) Scheme displaying the structure of HA-NONO expression constructs. RRM1, RNA recognition motif 1; RRM2, RNA recognition motif 2; NOPS, NonA/paraspeckle domain. (**B**) Immunoblots detecting HA-NONO variants and endogenous NONO. Vinculin, loading control; mock, non-transfected control. (**C, D**) Imaging (C) and quantitation (D) of HA-NONO variants and fibrillarin ±etoposide. Mock, non-transfected control. White box, zoomed image shown in Figure 1. Each dot represents % of cells with pan-nuclear signals as average from one acquisition. *, p-value <0.05; **, p-value <0.001; two-tailed t-test. Error bar, mean ±SD. Representative images are shown.

Supplementary Figure 4

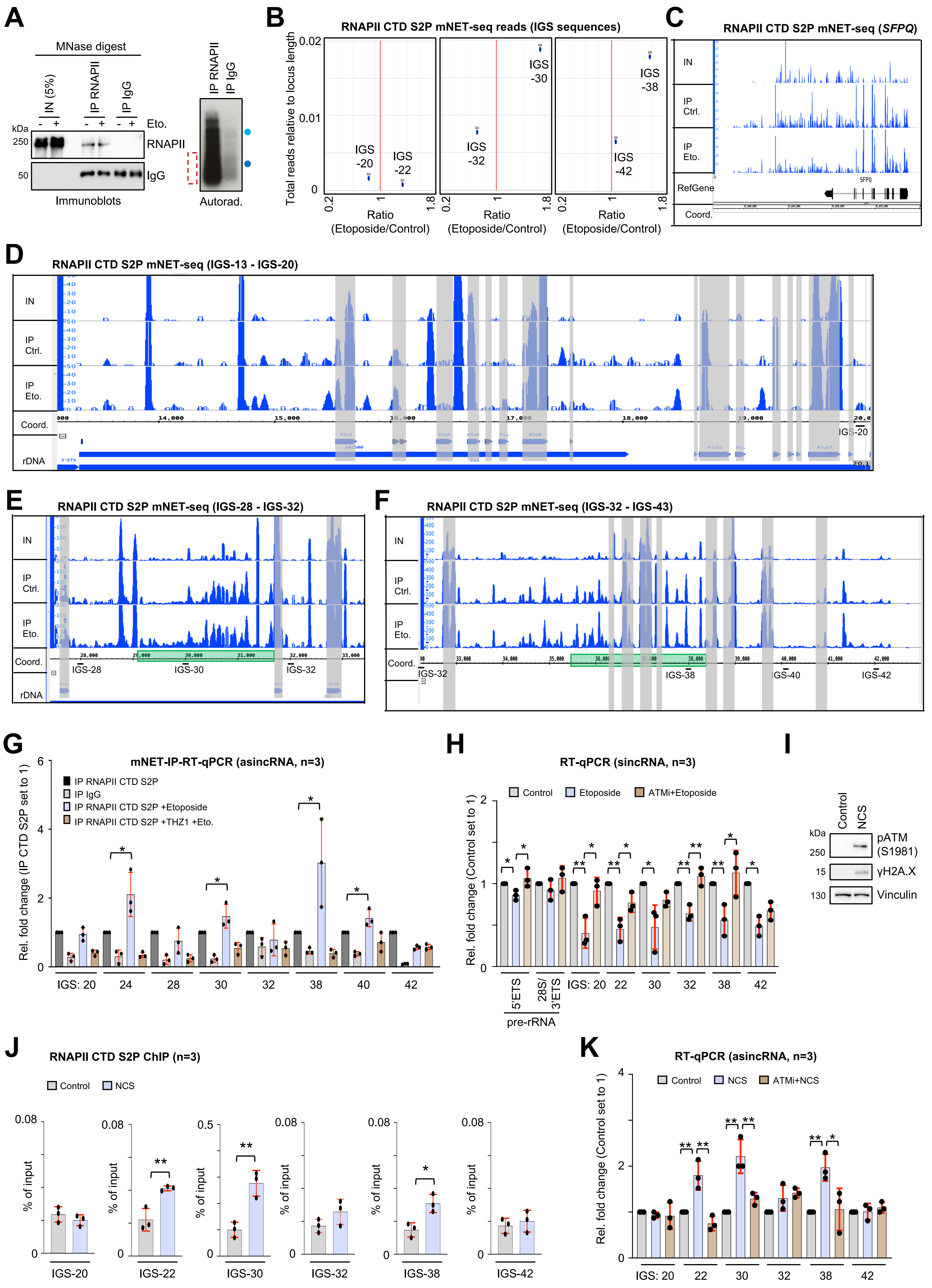

Supplementary Figure 4. Quality control for mNET-seq in U2OS cells. (**A**) Immunoblots detecting total RNAPII in input (IN, 5% of digest) and mNET-IP samples (10% material) that were immunoselected using RNAPII antibody from micrococcal nuclease (MNase)-digested samples ±etoposide; IgG, control (left). Autoradiograph detecting end-labeled transcripts upon immunoprecipitation (10% material) and PAGE separation (right). Blue dots, xylene cyanol/ bromophenol blue, size markers; red box, size-selected region. (**B**) Scatter plot displaying the number and relative abundance of mNET-seq reads ±etoposide (pairwise comparison of selected IGS sequences). (**C-F**) mNET-seq browser tracks for protein-coding gene *SFPQ* (C) or nucleolar IGS consensus regions 13-20 (D), 28-32 (E), and 32-43 (F) from inputs (IN, merged) or after immunoprecipitation (IP) with CTD S2P-selective antibody ±etoposide. Grey, *Alu* element; green, region of induction. IGS probe positions are not in scale. (**G**) RT-qPCR of transcripts associated with CTD S2P after immunoprecipitation (mNET-IP) ±etoposide or after preincubation with THZ1; IgG, control. (**H**) RT-qPCR assessing transcript levels from total RNA ±etoposide or pretreatment with ATM inhibitor. (**I**) Immunoblots detecting phospho-(p)ATM and γH2A.X ±Neocarzinostatin (NCS) treatment. Vinculin, loading control. (**J**) CTD S2P ChIP with site-specific primers. (**K**) RT-qPCR assessing transcript levels from total RNA ±NCS or pretreatment with ATM inhibitor. *, p-value <0.05; **, p-value <0.001; two-tailed t-test. Error bar, mean ±SD. n=number of biological replicates.

Supplementary Figure 5

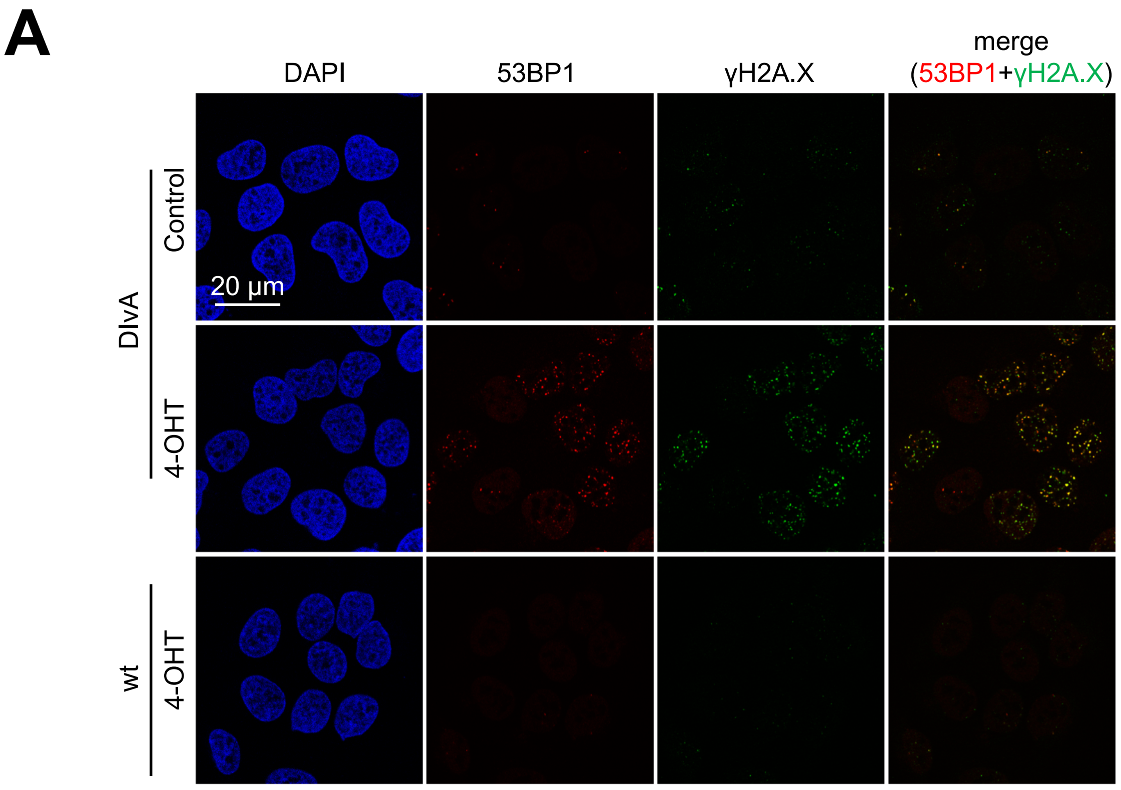

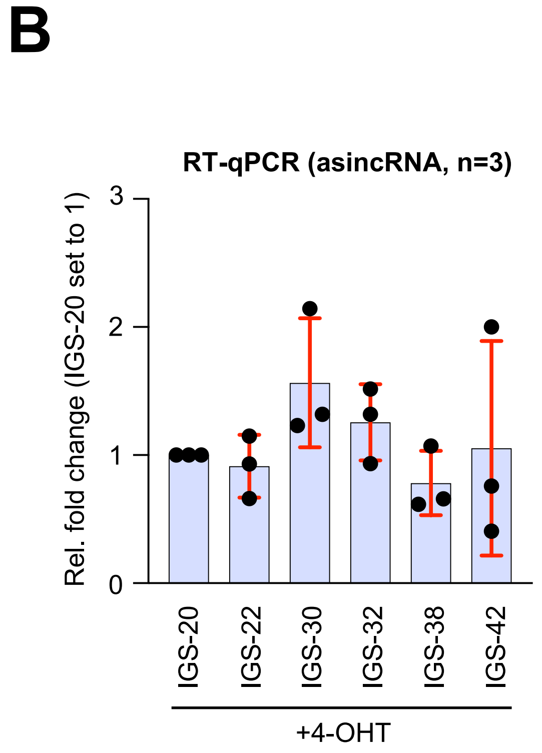

Supplementary Figure 5. DIvA system for the assessment of asincRNA. (**A**) Imaging of 53BP1 and γH2A.X in wild type (wt) or DIvA U2OS cells ±4-OHT. (**B**) RT-qPCR assessing transcript levels after incubation of DIvA U2OS cells with 4-OHT. *, p-value <0.05; **, p-value <0.001; two-tailed t-test. Error bar, mean ±SD. Representative images are shown. n=number of biological replicates.

Supplementary Figure 6

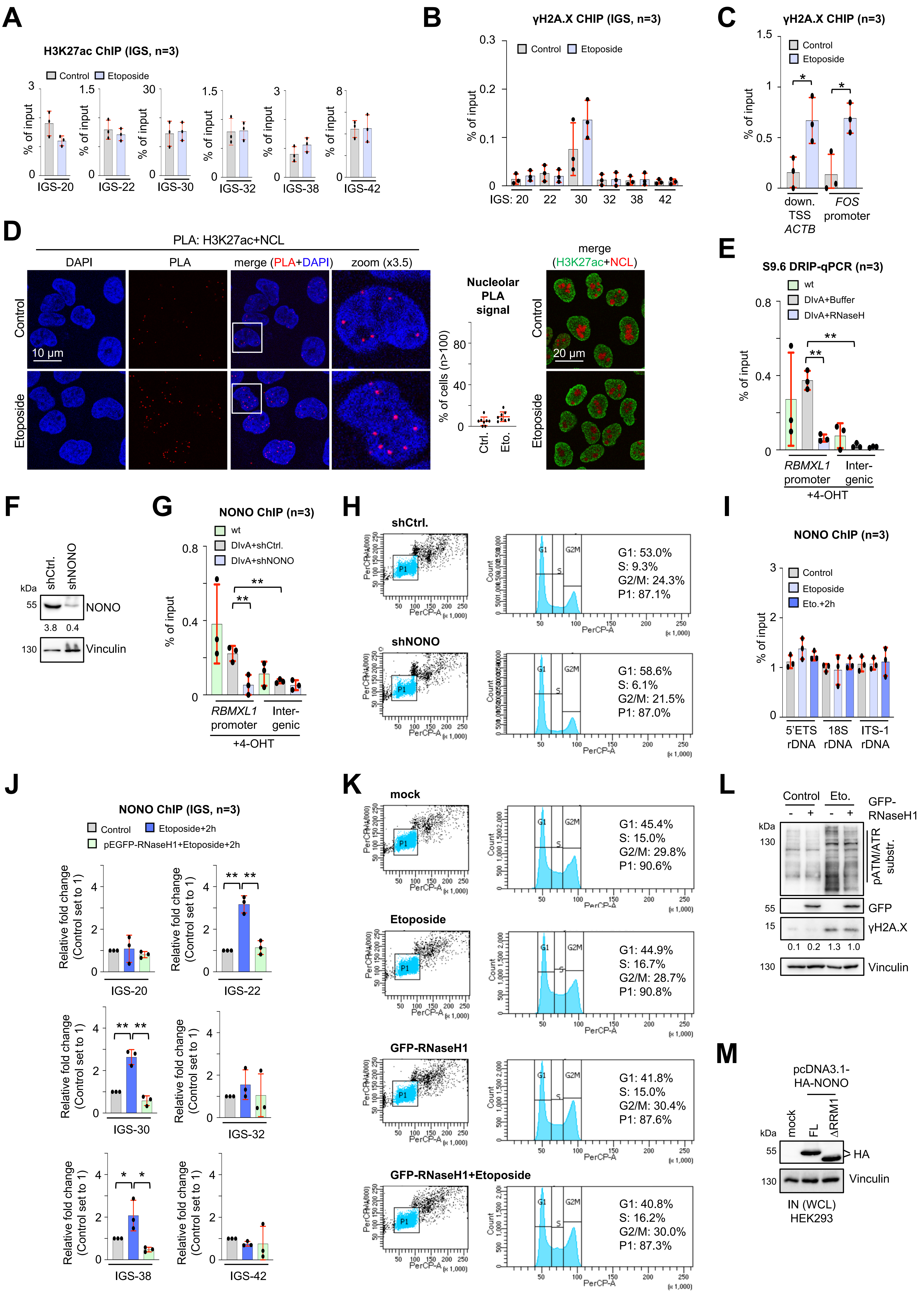

Supplementary Figure 6. Quality control for the assessment of chromatin marks, R-loops, NONO depletion and NONO ChIP in U2OS cells. (**A-C**) H3K27ac ChIP (A) or γH2A.X ChIP (B, C) using site-specific primers. (**D**) Imaging and quantitation of proximity ligation assay (PLA) signals (left) or indirect immunofluorescence signals (right) for H3K27ac/NCL. Each dot represents one acquisition. n, number of cells. (**E**) Quantitative PCR of DNA immunopurified from DNA-RNA hybrids (DRIP-qPCR) upon incubation with 4-OHT and recombinant RNaseH or buffer using S9.6 antibody and region-specific primers. (**F**) Immunoblots detecting NONO upon shRNA transduction. Vinculin, loading control. (**G**) ChIP analysis of NONO occupancy using site-specific primers upon incubation with 4-OHT and shRNA transduction. (**H**) Cell cycle analysis by fluorescence-activated cell sorting (FACS) ±NONO depletion. Gating (P1) of propidium iodide-positive, viable, non-duplet cells (left) and stratification for cell cycle phase (right). PerCP-A/ PerCR, forward/sideward scatter. A representative experiment is shown. (**I, J**) ChIP analysis of NONO occupancy using site-specific primers ±etoposide or after chase (+2h) (I) or after overexpression of GFP-RNaseH1 (J). 5'ETS, 5'external transcribed spacer; ITS-1, internal transcribed spacer-1. (**K**) Cell cycle analysis by FACS ±ectopic expression of GFP-RNaseH1/±etoposide. Gating, stratification and scatter as in (H). A representative experiment is shown. (**L**) Immunoblots detecting phosphorylated substrates of ATM/ATR kinases (pATM/ATR substr.), GFP and γH2A.X ±GFP-RNaseH1 overexpression/etoposide. Vinculin, loading control. (**M**) Immunoblots detecting HA-NONO variants FL and ΔRRM1 from HEK293 whole cell lysate (WCL) inputs (IN). Vinculin, loading control; mock, non-transfected control. *, p-value <0.05; **, p-value <0.001; two-tailed t-test. Error bar, mean ±SD. Representative images are shown. n=number of biological replicates.

Supplementary Figure 7

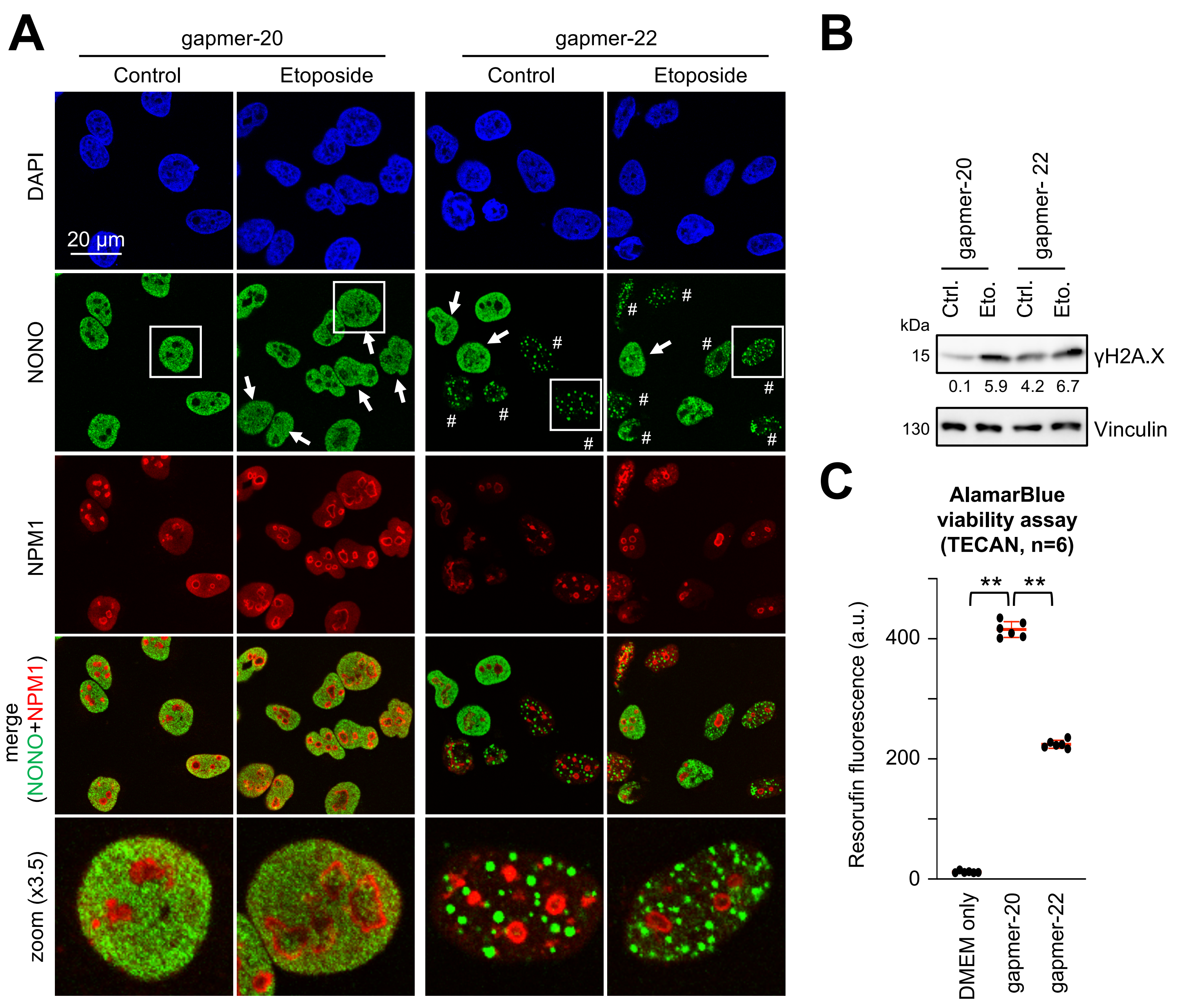

Supplementary Figure 7. Impact of IGS-selective gapmers on NONO localisation, DNA damage and viability in U2OS cells. (**A**) Imaging of NONO and NPM1 upon transfection of gapmers ±etoposide. Arrowhead, pan-nuclear NONO signal; #, nucleoplasmic NONO foci. (**B**) Immunoblots detecting γH2A.X ±etoposide. Vinculin, loading control. (**C**) AlamarBlue viability assay detecting resorufin signals as arbitrary units (a.u.) in U2OS cell supernatants upon transient transfection with selective gapmers. Each dot represents one measurement. Representative images are shown. *, p-value <0.05; **, p-value <0.001; two-tailed t-test. Error bar, mean ±SD. n=number of biological replicates.

Supplementary Figure 8

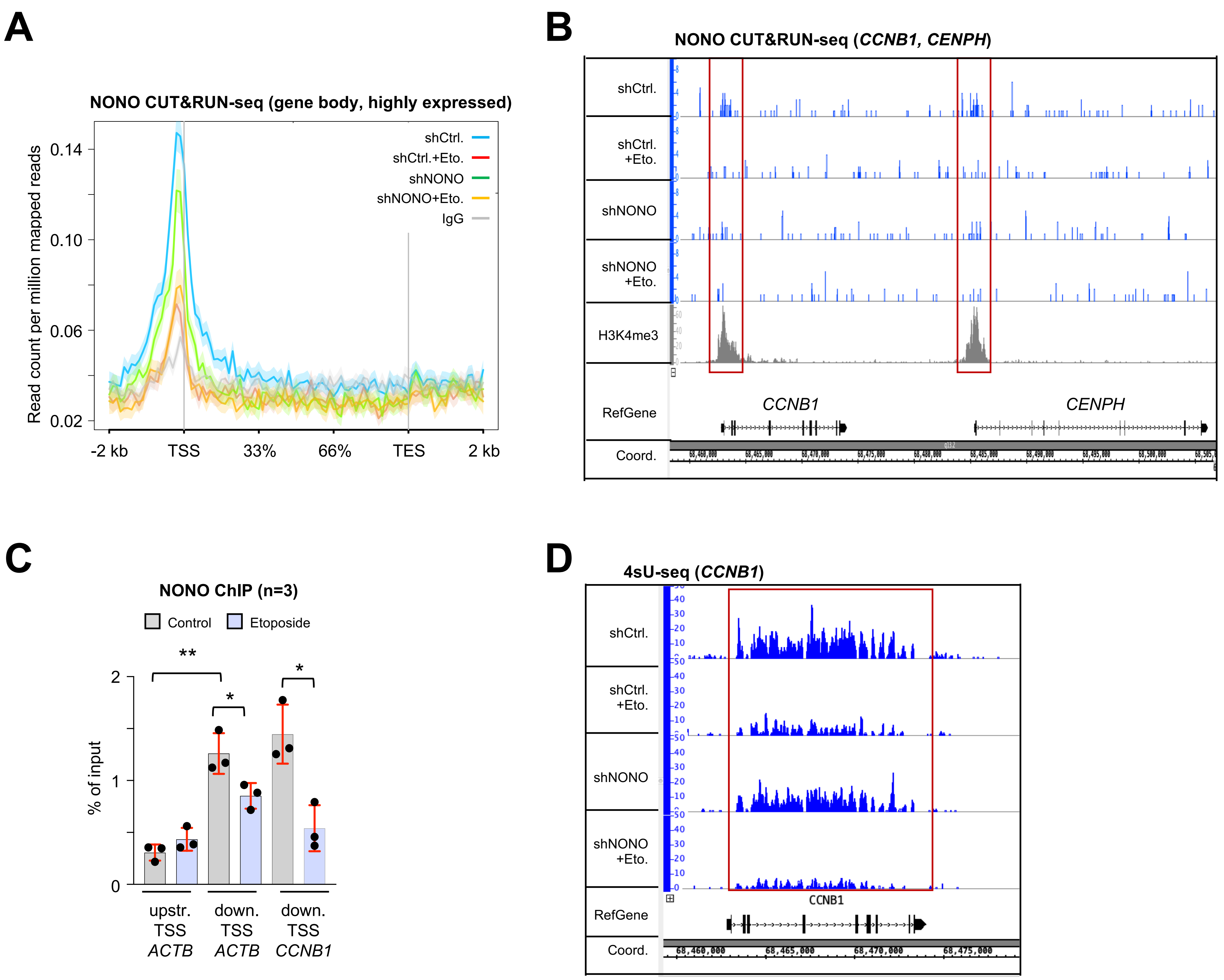

Supplementary Figure 8. DNA damage reduces NONO chromatin occupancy at TSSs of highly expressed genes and attenuates RNAPII activity in U2OS cells. (**A**) CUT&RUN-seq metagenes displaying NONO chromatin occupancy across the gene body of highly expressed genes ±NONO depletion/etoposide. (**B**) Browser tracks of NONO and histone H3 lys-4 tri-methylation (H3K4me3) CUT&RUN-seq ±NONO depletion/etoposide. Red box/H3K4me3, promoter region. (**C**) ChIP analysis of NONO occupancy using site-specific primers ±etoposide. (**D**) Browser tracks of 4sU-seq ±NONO depletion/etoposide. Red box, repressed region. *, p-value <0.05; **, p-value <0.001; two-tailed t-test. Error bar, mean ±SD. Representative images are shown. n=number of biological replicates.

Supplementary Figure 9

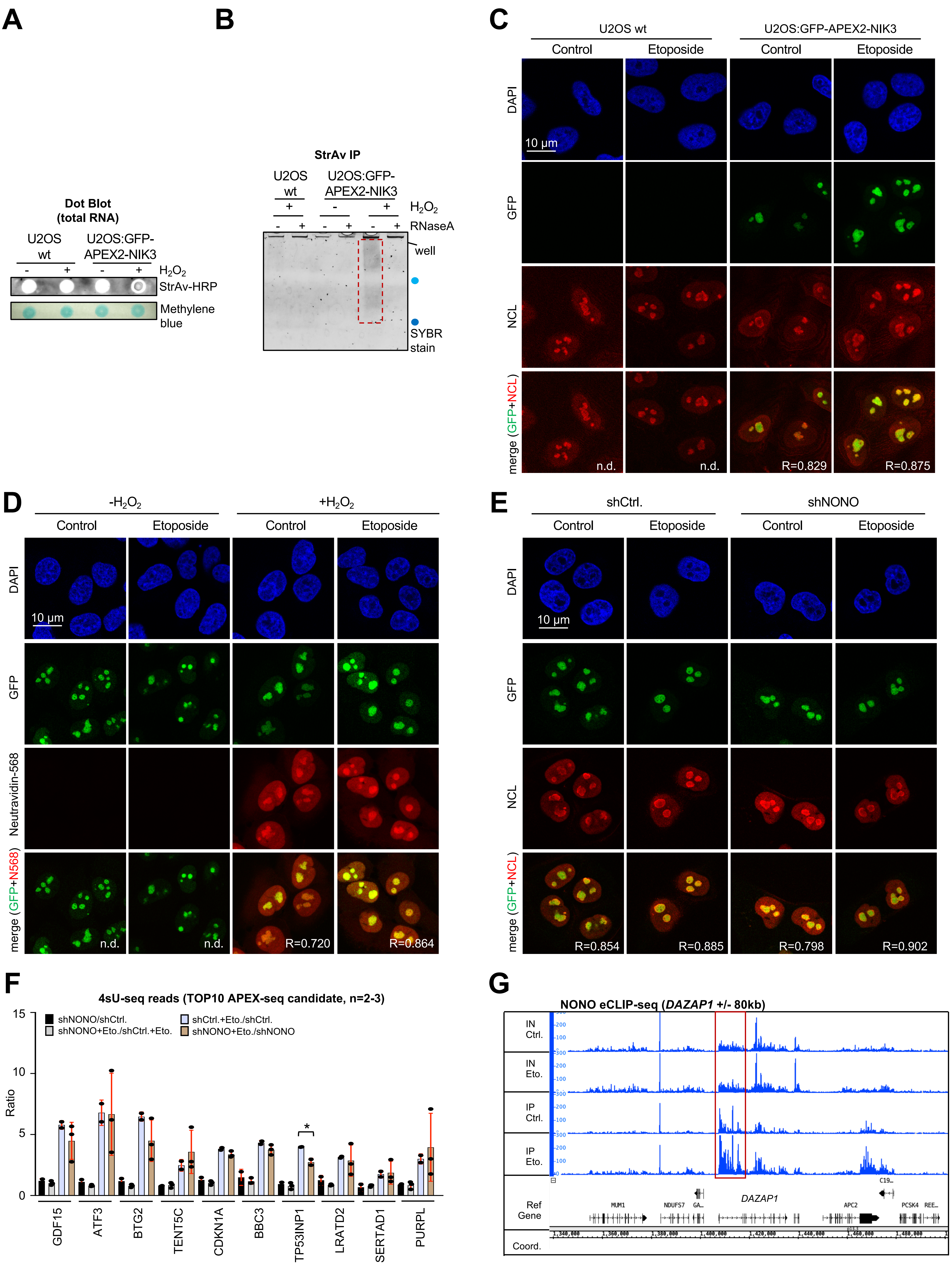

Supplementary Figure 9. Quality controls for APEX-seq and eCLIP-seq. (**A**) Dot blot analysis of total RNA from U2OS wild type or U2OS:GFP-APEX2-NIK3 cells using immunoblotting with a streptavidin-horseradish peroxidase (StrAv-HRP) probe ±hydrogen peroxide (H_2O_2). Methylene blue, loading control. (**B**) SYBR gold stain of RNA upon StrAv IP ±RNaseA digestion and PAGE separation from wt and U2OS:GFP-APEX2-NIK3 cells incubated ±H_2O_2. Blue dots, xylene cyanol/ bromophenol blue, size markers; red box, biotinylated material. (**C, D**) Imaging of GFP and NCL (C) in wild type and U2OS:GFP-APEX2-NIK3 cells ±etoposide or GFP and neutravidin-568 (D) in U2OS:GFP-APEX2-NIK3 cells ±etoposide and ±H_2O_2. (**E**) Imaging of GFP and NCL in U2OS:GFP-APEX2-NIK3 cells ±NONO depletion/etoposide. R=Pearson correlation; n.d., not detected. Representative images are shown. (**F**) Plot showing ratios of 4sU-seq reads for top 10 APEX-seq candidates ±NONO depletion/etoposide. *, p-value <0.05; **, p-value <0.001; two-tailed t-test. Error bar, mean ±SD. (**G**) Browser tracks depicting NONO eCLIP-seq reads for the DAZAP1 transcript. Red box, increased binding. n=number of biological replicates.

Supplementary Figure 10

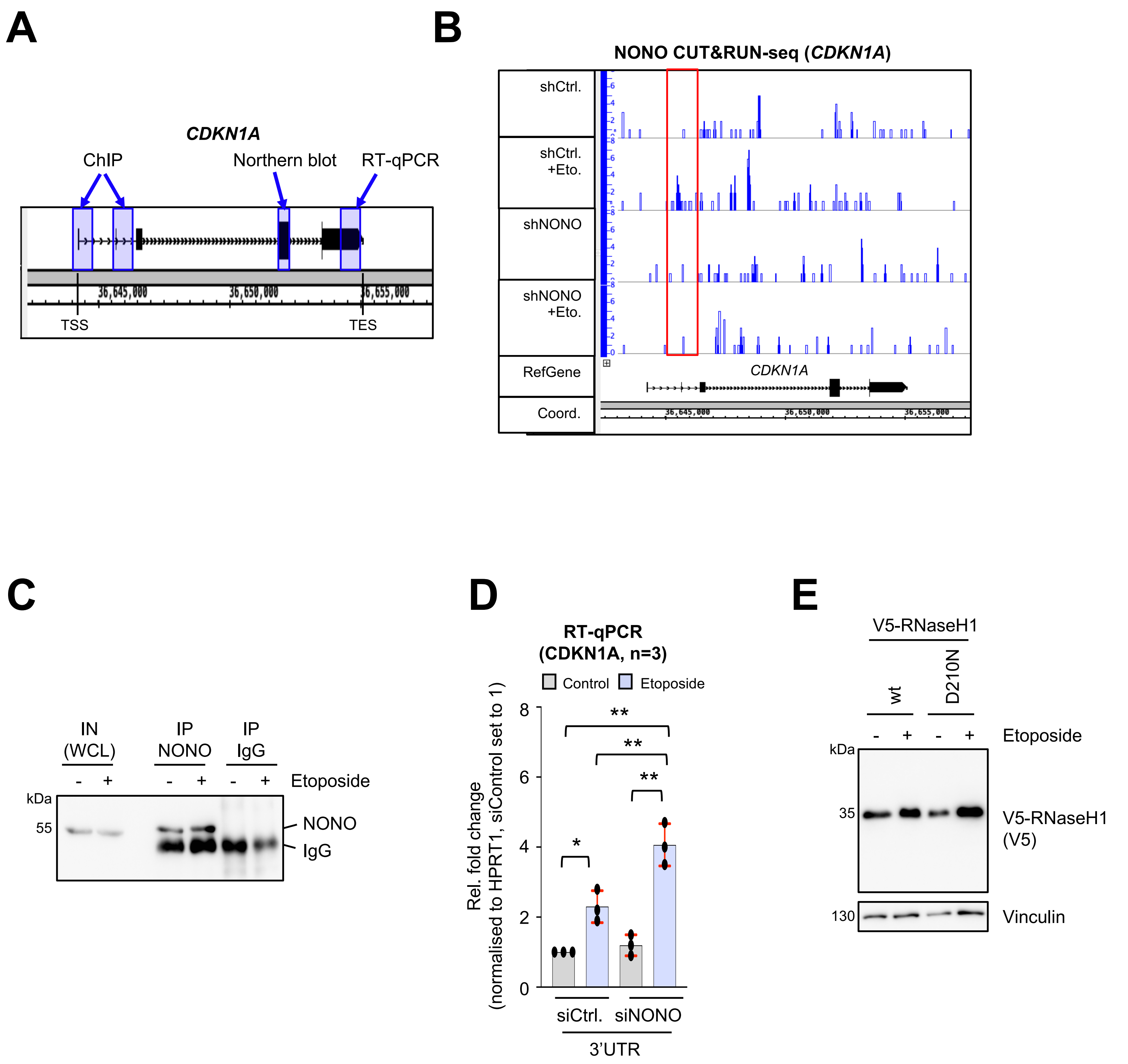

Supplementary Figure 10. Quality controls for the detection of CDKN1A transcripts and R-loops in U2OS cells. (**A**) Scheme displaying oligonucleotide targeting regions for ChIP, RT-qPCR and northern blot hybridisation at the *CDKN1A* locus. (**B**) Browser tracks depicting NONO CUT&RUN-seq reads for *CDKN1A* ±NONO depletion/etoposide. Red box, region of increase. (**C**) Immunoblot detecting NONO in input (IN) or upon immunoprecipitation (IP) from of whole cell lysates (WCL) of cells incubated ±etoposide. IgG, immunoglobulin, control IP. (**D**) RT-qPCR using site-specific primers ±NONO depletion/etoposide. (**E**) Immunoblot detecting V5-tagged RNaseH1 wt or D210N mutant ±etoposide. Vinculin, loading control. *, p-value <0.05; **, p-value <0.001; two-tailed t-test. Error bar, mean ±SD. n=number of biological replicates.

Supplementary Figure 11

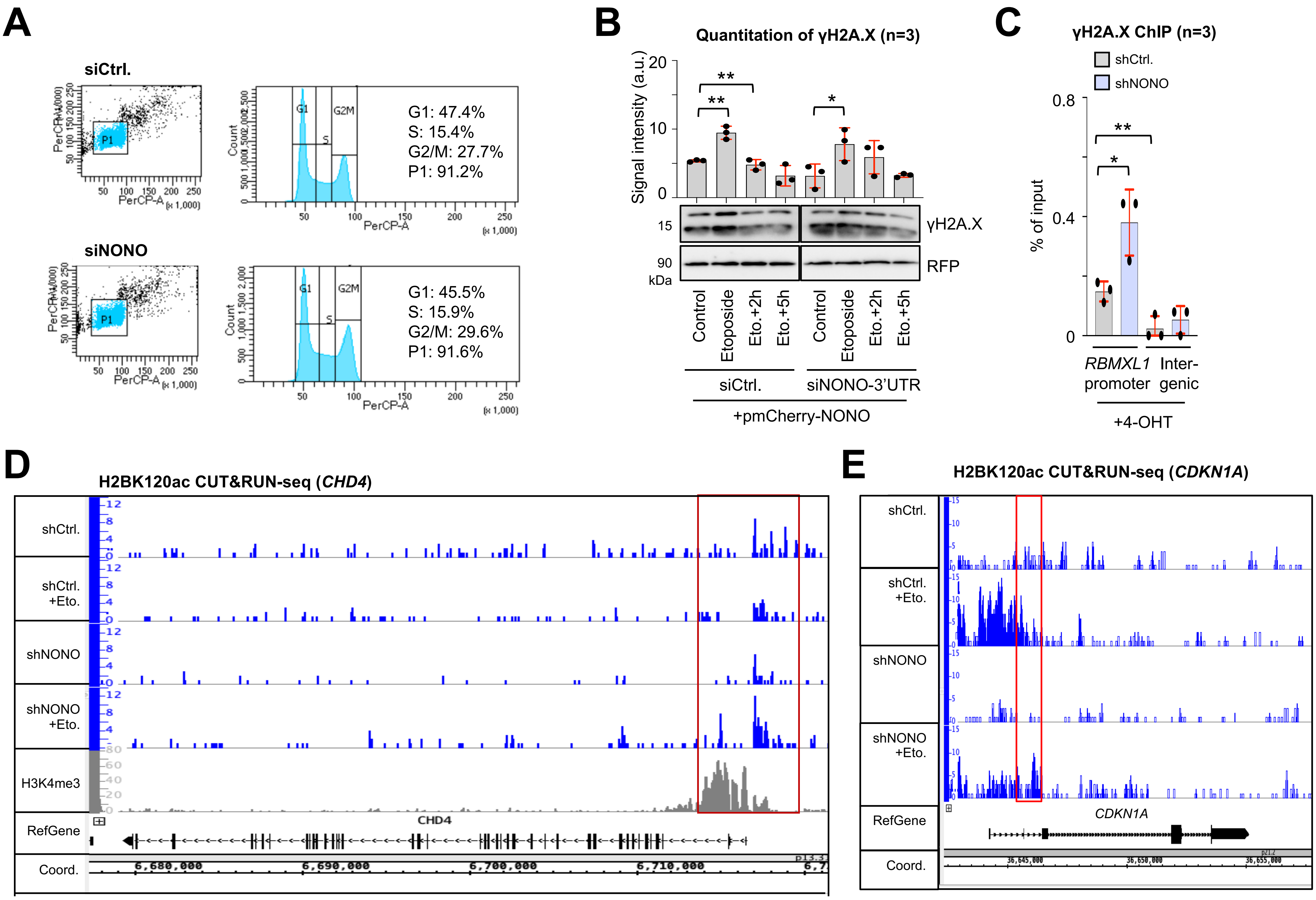

Supplementary Figure 11. Defects in DSB signaling upon depletion of NONO in U2OS cells. (**A**) Cell cycle analysis by fluorescence-activated cell sorting (FACS) upon siRNA transfection. Gating (P1) of propidium iodide-positive, viable, non-duplet cells (left) and stratification for cell cycle phase (right). PerCP-A/ PerCR, forward/sideward scatter. A representative experiment is shown. (**B**) Quantitation (top) and immunoblots (bottom) detecting γH2A.X and RFP ±NONO depletion/etoposide and transient expression of mCherry-NONO. a.u., arbitrary units. (**C**) ChIP analysis of γH2A.X occupancy using site-specific primers upon 4-OHT incubation ±NONO depletion. (**D, E**) Browser tracks of histone H2B lys-120 acetylation (H2BK120ac) CUT&RUN-seq ±NONO depletion/etoposide for *CHD4* (D) and *CDKN1A* (E). Red box/H3K4me3, promoter region. *, p-value <0.05; **, p-value <0.001; two-tailed t-test. Error bar, mean ±SD. n=number of biological replicates.

Supplementary Table 1. Primer pairs used for site-directed mutagenesis. Primers were custom made (Sigma).

**Primer**	**Sequence (5'-3')**
ΔRRM1-FWD	GCCTGCCATAGTGCATCCCTTAC
ΔRRM1-REV	TTGGGTGAAGGTCTTCTCTCCTGGTTTTC
ΔC-TER-FWD	TAACTCGAGCATGCATCTAGAGGGC
ΔC-TER-REV	CGCATCAGGGAAGGTTCCC
RRM1-FWD	TAACTCGAGCATGCATCTAGAGGGC
RRM1-REV	AAAGCGCACACGCAGCTGC

Supplementary Table 2. siRNA used for RNA interference.

**siRNA**	**Sequence (5’-3’)**	**Supplier, code**
siCtrl.	smart pool	Dharmacon, D-001810-01
siNONO	Smart pool	Dharmacon, L-007756-01
siNONO-3’UTR	GGAGUAUGCUGGAGGCAGAdTdT	Sigma, custom

Supplementary Table 3. Antibodies used in this study.

**Primary antibody**	**Species**	**Supplier, code**
IgG control	rabbit	Proteintech, 30000-0-AP
Anti-NONO	rabbit	Proteintech, 11058-1-AP
Anti-PSPC1	rabbit	Proteintech, 16714-1-AP
Anti-SFPQ [EPR11874]	rabbit	Abcam, ab177149
Anti-NPM1 [FC82291]	mouse	Abcam, ab10530
Anti-NPM1 [7H10B9]	mouse	Invitrogen, MA5-17141
Anti-phospho-histone H2A.X (S139)	rabbit	Cell Signaling, 2577
Anti-phospho-histone H2A.X (S139) [3F2]	mouse	Abcam, ab22551
Anti-phospho-histone H2A.X (S139) [JBW301]	mouse	Millipore, 05-636
Anti-nucleolin [364-05]	mouse	Abcam, ab136649
Anti-fibrillarin	rabbit	Abcam, ab5821
Anti-vinculin	mouse	Sigma, V9131
Anti-HA tag [16B12]	mouse	BioLegend, 901502
Anti-HA tag	rabbit	Abcam, ab9110
Anti-RNA polymerase II RPB1	mouse	BioLegend, 920204
Anti-RNA polymerase II CTD repeat YSPTSPS	rabbit	Abcam, ab26721
Anti-phospho-RNA polymerase II CTD repeat YSPTSPS (S2)	rabbit	Abcam, ab5095
Anti-V5 tag	mouse	Thermo, R960-25
Anti-SPT5 [D-3]	mouse	Santa Cruz, sc133217

Anti-DNA-RNA-hybrid [S9.6]	mouse	Millipore, MABE1095
Anti-53BP1	rabbit	Novus, NB100-304
Anti-phospho-ATM (S1981) [EP1890Y]	rabbit	Abcam, ab81292
Anti-phospho-ATM/ATR substrate (S*Q) [D23H2/D69H5]	rabbit	Cell Signaling, 9607
Anti-histone H2BK120ac	rabbit	Active Motif, 39120
Anti-RFP tag [RF5R]	mouse	Thermo, MA5-15257
Anti-GFP tag	rabbit	Abcam, ab290
Anti-ATM [D2E2]	rabbit	Cell Signaling, 2873
Anti-H3K4me3	rabbit	Abcam, ab8580
Anti-H3K27ac	rabbit	Cell Signaling, 8173
Anti-TOPBP1	rabbit	Thermo, PA5-65178
Anti-TCOF1 [H-6]	mouse	Santa Cruz, sc-374536
Anti-TCOF1	rabbit	Sigma, HPA038237
Anti-XRCC4	rabbit	Abcam, ab97351
**Secondary antibody**	**Species**	**Supplier, code**
HRP-linked IgG	mouse	Cytivia, NA931
HRP-linked IgG	rabbit	Cytivia, GEHENA934
Alexa Fluor 546-linked IgG	rabbit	Thermo, A10040
Alexa Fluor 488-linked IgG	mouse	Thermo, A32766TR
Alexa Fluor 546-linked IgG	mouse	Thermo, A10036
Alexa Fluor 488-linked IgG	rabbit	Thermo, A21206
Alexa Fluor 568-linked IgG	mouse	Invitrogen, A-11031

Alexa Fluor 488-linked IgG	rabbit	Invitrogen, A-11034
Alexa Fluor 647-linked IgG	rabbit	Invitrogen, A-21245

Supplementary Table 4. Gapmers used for end-labeling, binding assays and transfections. All bases are DNA except ones preceded by 'm' like mU, which are 2'-hydroxy methylated RNA bases. *, phosphorothioate linkage. Gapmers were custom made (IDT).

**Gapmer**	**Sequence (5'-3')**
gapmer-20	mG*mU*mA*mG*mC*C*T*T*G*G*G*C*T*T*C*mU*mC*mU*mC*mC
gapmer-22	mC*mA*mG*mU*mG*G*C*T*C*A*C*G*T*C*T*mG*mU*mC*mA*mU

Supplementary Table 5. Adapters used for DSB ligation. Adapters were custom made (Sigma).

**BLISS adapter**	**Sequence (5’-3’)**
adapter A1	CATCACGC
adapter A2	GTCGTTCC
adapter A3	TGATGATC
adapter A4	ACGACATC

Supplementary Table 6. Primer pairs used for RT-qPCR and ChIP. Primers were custom made (Sigma).

**Primer**	**Sequence (5'-3')**
IGS20-fwd	GTAGCCTTGGGCTTCTCTCC
IGS20-rev	AGTTTTCAGCCCCAACACAC
IGS22-fwd	CAGTGGCTCACGTCTGTCAT
IGS22-rev	CGCCTGACTCCATTTCGTAT
IGS24-fwd	CCCGCGCACATAATAACTAA
IGS24-rev	AAATCACTCCTCACGGGAAC
IGS28-fwd	CCTTCCACGAGAGTGAGAAG
IGS28-rev	GACCTCCCGAAATCGTACAC
IGS30-fwd	GGTCTCTGCGTCTCGCTATC
IGS30-rev	TGAAGAATTCAGGCCTTGGT
IGS32-fwd	AAAAGCTGGCCGATCTGAAT
IGS32-rev	CGTCTGTTCAGCTATTTTGCAG
IGS38-fwd	CTCACAGAGGAAGGGAGCAC
IGS38-rev	AACAAGGGAGGGAGGAACTT
IGS40-fwd	TTCTCCTTGGTCAGGGGTTT
IGS40-rev	CAGGAAAGTCCCCAACAACA
IGS42-fwd	GCTTCTCGACTCACGGTTTC
IGS42-rev	CCGAGAGCACGATCTCAAAG
5‘ETS-fwd	GCCCCGGGGGAGGTAT
5‘ETS-rev	GAGGACAGCGTGTCAGC
18S-fwd	GTTGAACCCCATTCGTGATG

18S-rev	GGGACTTAATCAACGCAAGC
ITS-1-fwd	TGTGAAACCTTCCGACCCC
ITS-1-rev	GGGGTTGCCTCAGGCC
upstream-TSS-ACTB-fwd	CCCACCTGACAACCTCTCAT
upstream-TSS-ACTB-rev	CCCTTCTTGCTGCCTGTT
downstream-TSS-ACTB-fwd	CTCAATCTCGCTCTCGCTCT
downstream-TSS-ACTB-rev	CTCGAGCCATAAAAGGCAAC
downstream-TSS-CCNB1-fwd	ATCGCCCTGGAAACGCATTCT
downstream-TSS-CCNB1-rev	GCCAGCCTAGCCTCAGATTTA
RBMXL1-promoter-DSB-fwd	GATTGGCTATGGGTGTGGAC
RBMXL1-promoter-DSB-rev	CATCCTTGCAAACCAGTCCT
Intergenic-no DSB-fwd	ATTGGGTATCTGCGTCTAGTGAGG
Intergenic-no DSB-rev	GACTCAATTACATCCCTGCAGCT
HPRT1-fwd	AGATGTGATGAAGGAGATGG
HPRT1-rev	AATAGCTCTTCAGTCTGATAAAATC
GAPDH-fwd	AACCTGCCAAATATGATGAC
GAPDH-rev	AGGAAATGAGCTTGACAAAG
CDKN1A-3’UTR-fwd	CTTGAGTGGGGTTATCTCTG
CDKN1A-3’UTR-rev	CAGTAGAGGCTATGGACAGG
FOS-promoter-fwd	AGGTTTCCACGGCCTTTCC
FOS-promoter-rev	TTTCGCAGTTCCTGTCTCAGAG
5’ETS-pre-rRNA-fwd	CCAGTGGTTGTCGACTTG
5’ETS-pre-rRNA-rev	AACGACACACCACCGTTC
28S/3’ETS-pre-rRNA-fwd	GCGCTAAACCATTCGTAG

28S/3’ETS-pre-rRNA-rev	GAAGACGAACGGAAGGAC
CDKN1A-TSS-fwd	GAACAGGGTATGTGATCTGC
CDKN1A-TSS-rev	AGCAGGCTGTTAAAAGTCAC
CDKN1A-intron-fwd	GAAGTCAGTTCCTTGTGGAG
CDKN1A-intron-rev	GCAGAAACACCTGTGAACG

Supplementary Table 7. DNA probe used for northern blot hybridisation. Probe was custom made (Sigma).

**Probe**	**Sequence (5'-3')**
CDKN1A-exon	GCACAAGGGTACAAGACAGTGACAGGTCCACATGGTCTTCCTCTGCTGTC

Supplementary Table 8. Quasar-570-labeled probes used for RNA-FISH. Probes were custom made (IDT).

**Probe**	**Sequence (5'-3')**
IGS-22 (30 probes)	aaatgaaagaaaaggcac
	acagatatacaagaaaga
	catacatacatccatgca
	catacatacatacataca
	tccagataaatacgtaca
	gacagagtgagacccggt
	actgcactacagcctggg
	agtgacccaagatcgcac
	cctggcaggcggaggctg
	ggtggaagaattgcttga
	cagctactcgggaggctg
	ggggcaggcacctgtaac
	aaatggagtcaggcgccg
	cgtctctactgaaaatac
	ggccaacgtggtgaaacc
	aggagttcgagaccagcc
	cgtctgtcatcccgaggt
	cactgaacgcagtggctc
	tctaagaaatggtactgt
	tgttcccgtgagagtgat
	cacgtcacccataagtgt

	taactaactaaaatctct<br><br>ccacctcctcgcgcacat<br><br>agcaagagccaaactccg<br><br>cattgcactgtagcctgg<br><br>actccttgagtcccctag<br><br>gtgtcctctctgccgtag<br><br>cccttacgctcagaatga<br><br>ctgccacctttcgctgtg<br><br>agcctttaaaagcgcggc
